# Complexity and Local Specificity of the Virome Associated with Tospovirus-Transmitting Thrips Species

**DOI:** 10.1128/JVI.00597-21

**Published:** 2021-10-13

**Authors:** M. Chiapello, L. Bosco, M. Ciuffo, S. Ottati, N. Salem, C. Rosa, L. Tavella, M. Turina

**Affiliations:** a Institute for Sustainable Plant Protection, CNR, Turin, Italy; b Dipartimento di Scienze Agrarie, Forestali e Alimentari, University of Turin, Grugliasco, Turin, Italy; c Department of Plant Pathology and Environmental Microbiology, The Pennsylvania State University, University Park, Pennsylvania, USA; d Department of Plant Protection, School of Agriculture, The University of Jordan, Amman, Jordan; University of Texas Southwestern Medical Center

**Keywords:** entomovirus, thrips, virome

## Abstract

Frankliniella occidentalis (western flower thrips [WFT]) and Thrips tabaci (onion thrips [OT]) are insect species that greatly impact horticultural crops through direct damage and their efficient vectoring of tomato spotted wilt virus and iris yellow spot virus. In this study, we collected thrips of these species from 12 field populations in various regions in Italy. We also included one field population of Neohydatothrips variabilis (soybean thrips [ST]) from the United States. Total RNA data from high-throughput sequencing (HTS) were used to assemble the virome, and then we assigned putative viral contigs to each thrips sample by real-time reverse transcription-quantitative PCR (qRT-PCR). Excluding plant and fungal viruses, we were able to identify 61 viral segments, corresponding to 41 viruses: 14 were assigned to WFT, 17 to OT, and 1 to ST; 9 viruses could not be assigned to any species based on our stringent criteria. All these viruses are putative representative of new species (with only the exception of a sobemo-like virus that is 100% identical to a virus recently characterized in ST) and some belong to new higher-ranking taxa. These additions to the viral phylogeny suggest previously undescribed evolutionary niches. Most of Baltimore’s classes of RNA viruses were present (positive- and minus-strand and double-stranded RNA viruses), but only one DNA virus was identified in our collection. Repeated sampling in a subset of locations in 2019 and 2020 and further virus characterization in a subset of four thrips populations maintained in the laboratory allowed us to provide evidence of a locally persistent thrips core virome that characterizes each population.

**IMPORTANCE** Harnessing the insect microbiome can result in new approaches to contain their populations or the damage they cause vectoring viruses of medical, veterinary, or agricultural importance. Persistent insect viruses are a neglected component of their microbiota. In this study, for the first time, we characterize the virome associated with the two model systems for tospovirus-transmitting thrips species, of utmost importance for the direct and indirect damage they cause to a number of different crops. The thrips virome characterized includes several novel viruses, which in some cases reveal previously undescribed clades. More importantly, some of the viruses we describe are part of a core virome that is specific and consistently present in distinct geographical locations monitored over the years, hinting at a possible mutualistic symbiotic relationship with their host.

## INTRODUCTION

Thrips are taxonomically included in the order Thysanoptera. The order includes ca. 7,000 described species organized in two suborders: Tubulifera and Terebrantia (1). Thrips include species with different feeding behaviors: half of the described species feed on fungi, whereas the rest is mostly characterized by phytophagous behavior and feed on leaves, flowers and fruits; a few species are predators of other small arthropods (2). Most of the plant pest species are in the family Thripidae, which includes ca. 1,700 species. Phytophagous thrips can cause direct and indirect damage to the plants they feed on: indirect damage is greatly enhanced by their ability to transmit tospoviruses (genus *Orthotospovirus*, family *Bunyaviridae*). Currently, 14 species of thrips have been shown to transmit tospoviruses (3, 4). *Tospoviridae* are likely insect-infecting viruses that adapted to a plant host (5). The origin of the association between thrips and tospoviruses is not clear (6).

The two most important thrips species in the Mediterranean basin are Frankliniella occidentalis (Pergande) (western flower thrips [WFT]) and Thrips tabaci (Lindeman) (onion thrips [OT]) (7). WFT is native to the western United States and was first reported in the Mediterranean Basin in the late 1980s, but it is now present worldwide and is considered the most efficient vector of tomato spotted wilt virus (TSWV) and impatiens necrotic spot virus (INSV) in the Mediterranean Basin (7, 8). OT is instead native to the eastern Mediterranean, but it is now spread worldwide; in the Mediterranean basin, OT is now an efficient vector of iris yellow spot virus (IYSV). TSWV, INSV, and IYSV are endemically present in the Mediterranean Basin and Europe, and TSWV causes major loss in a number of horticultural crops, particularly because of resistance breaking strains in pepper and tomato (9). This necessitates new approaches in integrated pest management (IPM): in this regard, specific attention has been paid to the possibility of altering the microbiome associated with insects as a new tool for crop pest management (10).

For insect vectors of plant pathogens, an approach that does not put selective pressure on the insect population, but only on its vectoring competence, can be of great interest: a recent successful case study is that of the artificial introduction of a new *Wolbachia* strain to the brown planthopper Nilaparvata lugens (Stål), which resulted in the reduction of rice ragged stunt virus transmission (11). Other approaches can be envisioned based on the exploitation of the microbiome: specific virus-virus interactions could also be at the base of interference with vectoring ability, and synergistic or antagonistic relationships between resident/persistent insect viruses and the plant viruses they vector could indeed provide a new approach to contain tospovirus spread. In fact, viruses as elements of insect microbiomes are only minimally studied (12). After recent groundbreaking work showing the vast diversity of viruses present in insects and invertebrates (13–15), the importance of the insect microbiomes for virus evolution and biology is becoming apparent, but they are greatly understudied in their biological effects on the host. In this respect, up until very recently, viromes associated with thrips among the most important vectors of plant diseases have also been neglected: a list of viruses associated with Neohydatothrips variabilis (Beach) (soybean thrips [ST]), vector of soybean vein necrosis virus, has been only recently provided (16).

The purpose of this study was to characterize for the first time the virome associated with the two most efficient vectors of tospoviruses, WFT and OT, in the Mediterranean area, with an emphasis on their variability according to species, geographical location, and recurrence in different sampling efforts carried out between 2018 and 2020.

## RESULTS

### Taxonomic analysis of the meta-transcriptomes.

For each library, more than 100 million 150-bp paired-end reads were retrieved.

**FIG 1:**
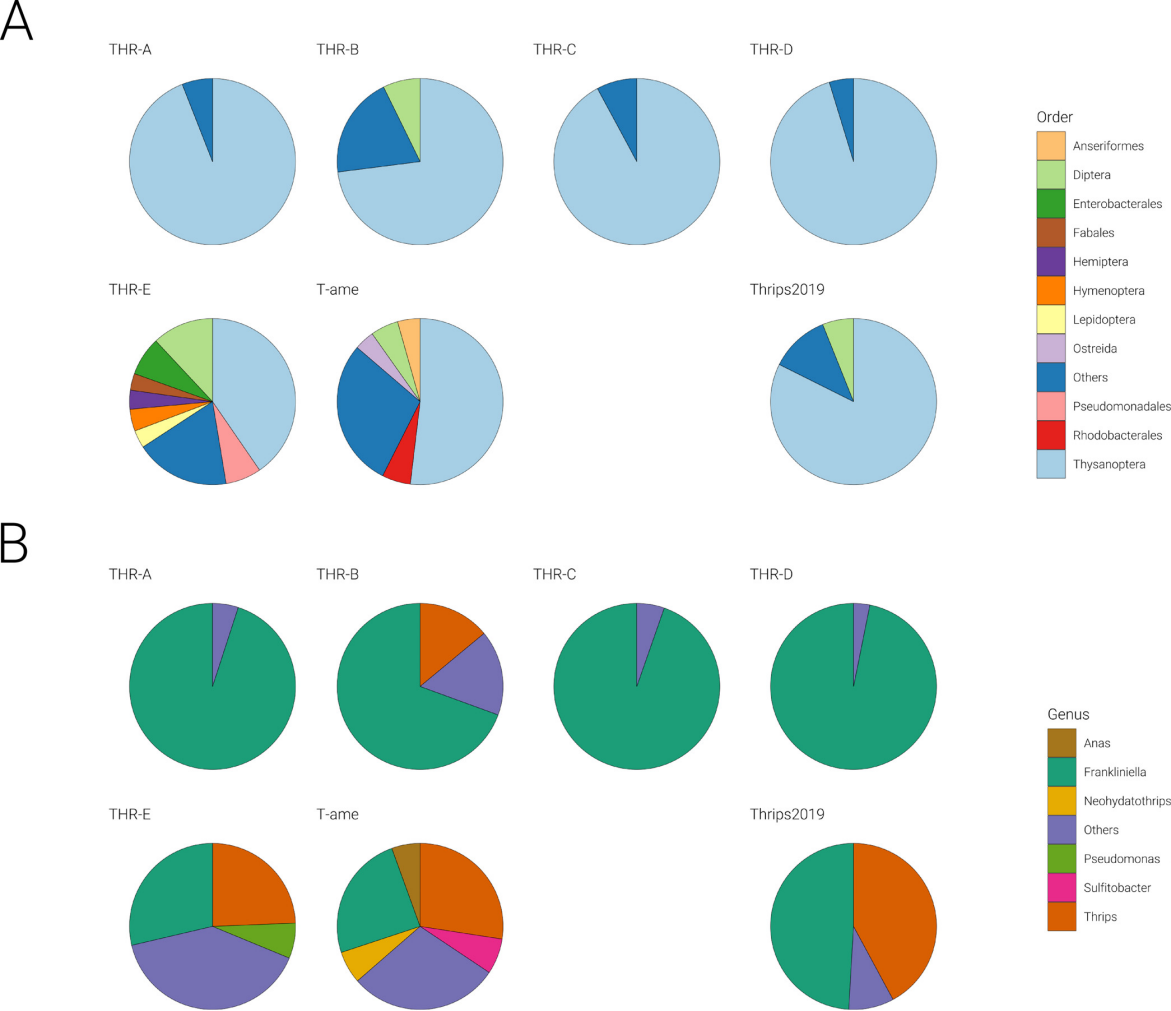
Pie charts represent the distribution of selected taxonomic groups across all pools, reporting the number of reads for each genus with more than 100,000 reads. (A) Number of reads for each order; (B) number of reads for each genus.

In order to check the taxonomic complexity of the samples, we performed a metatranscriptomic analysis using Kraken2. Results, processed with Pavian, show the sample composition of each library at different taxonomic levels: domain (data not shown), order (Fig. 1A), phylum (data not shown), and genus (Fig. 1B). At higher taxonomic ranks, all libraries (THR-A, THR-B, THR-C, THR-D, THR-E, T-ame, and Thrips2019) showed a clear prevalence (>90%) of Eukaryota. The lower taxonomic rank genus showed that in three libraries (THR-A, THR-C, and THR-D), the majority of sequences belong to *Frankliniella*, as expected. In the remaining libraries (THR-B, THR-E, T-ame, and Thrips2019), there is a mix of *Frankliniella* and *Thrips* genera, even if assignment of reads belonging to *T. tabaci* to the genus *Thrips* might be underestimated since only the Thrips palmi (Karny) genome is available in databases, and therefore, assignment of contigs/reads to *F. occidentalis* could be due to conservation of some sequences between the two genera and lack of a direct hit for *T. tabaci*. Moreover, the library THR-E contains many sequences that could not be assigned to thrips but are instead assigned to other genera, mostly of insects but also bacteria. In T-ame the genus *Neohydatothrips*, as expected, is also present. Referring to the order rank (Fig. 1A), two libraries (THR-E and T-ame) out of seven are more heterogeneous, including some other insect orders, among which are dipteran and hymenopteran (likely from some parasitoid insects).

### Viruses associated with thrips metatranscriptomic samples.

We discuss here all the viruses discovered over the years as true thrips viruses (confirmed in multiple instances in individual thrips checked for their taxonomic assignment); to be more conservative, the other viruses with less certain host assignment (or not reconfirmed in multiple assays on distinct individual thrips) are deposited in GenBank as “insect metagenomic” viruses, although some of them could indeed be thrips viruses.

**FIG 2:**
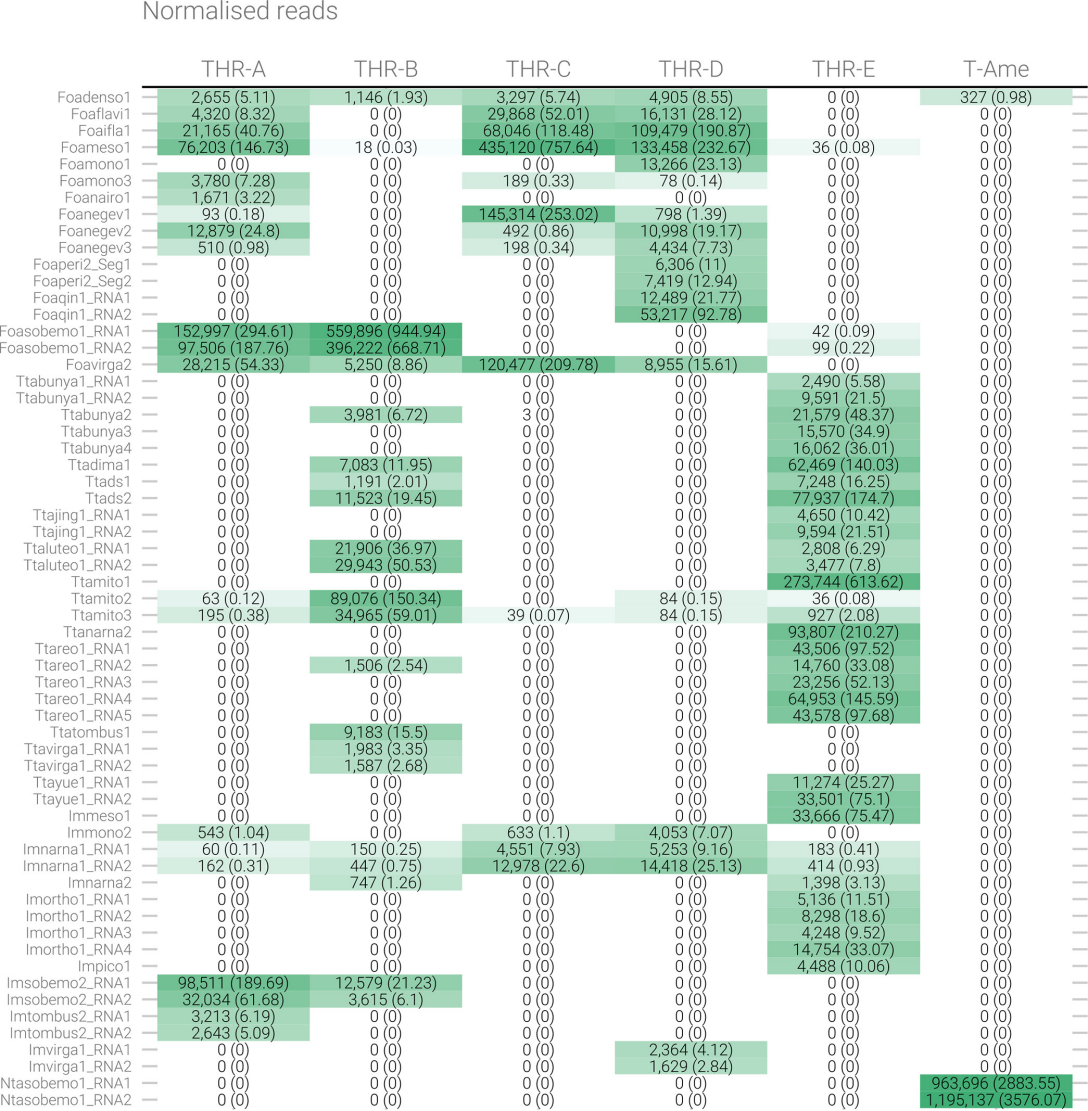
Heat map table showing the number of reads mapping on the identified viruses in each library. Numbers in parentheses correspond to normalized reads per kilobase per million mapped reads (RPKM). The intensity of the green color reflects the number of reads.

Overall, 95 viral contigs were identified (Tables 1 and 2), among which 13 were likely fungal viruses (based on their high identity percentages with confirmed mycoviruses present in databases), 17 were plant viruses (also based on their high identity percentages with confirmed viruses present in the databases), and 4 were likely endogenous viral elements in the host genome (positive PCR on DNA) (Table 1); 16 were uncertain insect host viral contigs (likely entomoviruses based on the first BLAST hit in databases), and 45 were confirmed thrips viral contigs corresponding to 32 viruses (some viral genomes are multisegmented) (Table 2). Within the 32 viruses with confirmed hosts, 14 infect WFT, 17 infect OT, and 1 infects the ST. Entomovirus contigs are reported in Fig. 2, where number of reads mapping to each contig and the normalized values are displayed (Fig. 2).

**TABLE 1:** List of putative plant, fungal and endogenized viral contigs discovered applying our bioinformatic pipeline to RNA sequencing data of thrips samples^*a*^

Contig	Length (no. of bases)	Accession no.	NCBI BLASTx first hit	Identity^*b*^	Origin
T-Ame_DN12303	3,216	QBX90567	Cucumber mosaic virus	100*	Plant virus
THR-B_DN28487	1,996	AOX21989	Iris yellow spot virus	100*	Plant virus
THR-D_DN18945	7,762	APG79622	Tomato spotted wilt tospovirus	99*	Plant virus
THR-E_DN20119	1,556	AWK67805	Iris yellow spot virus	99*	Plant virus
THR-E_DN20826	1,091	AWK67804	Iris yellow spot virus	99*	Plant virus
THR-E_DN23926	3,648	ACJ04669	Iris yellow spot virus	99*	Plant virus
THR-B_DN30354	8,873	YP_009241381	Iris yellow spot virus	99*	Plant virus
T-Ame_DN11632	3,025	CCQ26876	Cucumber mosaic virus	99*	Plant virus
THR-A_DN12663	1,489	YP_009551627	Melon partitivirus	64	Plant virus
THR-A_DN9251	1,541	YP_009551628	Melon partitivirus	41	Plant virus
THR-E_Contig1	2,488	QED45151	Leek yellow stripe virus	96*	Plant virus
THR-E_DN24091	10,357	AGG18220	Leek yellow stripe virus	89	Plant virus
THR-D_DN16018	2,950	QPZ88447	Tomato spotted wilt tospovirus	95*	Plant virus
THR-C_DN21959	8,918	AKM21265	Tomato spotted wilt tospovirus	100*	Plant virus
THR-A_DN26458	2,851	ASU87377	Bell pepper alphaendornavirus	100*	Plant virus
THR-A_Contig1	9,812	AJF48474	Bell pepper alphaendornavirus	100*	Plant virus
THR-C_DN28521	14,708	AYR00620	Bell pepper alphaendornavirus	98*	Plant virus
T-Ame_DN13641	1,013	NP_620728	Ustilago maydis virus H1	70	Fungal virus
T-Ame_DN4615	3,117	NP_620728	Ustilago maydis virus H1	48	Fungal virus
THR-B_DN29920	1,119	YP_009508064	Heterobasidion partitivirus 8	35	Fungal virus
THR-E_DN21437	3,110	QDH89606	*Mitovirus* sp.	65	Fungal virus
THR-E_DN23537	27,80	QDB75006	Acremonium sclerotigenum ourmia-like virus 1	60	Fungal virus
THR-E_DN24038	2,138	BBZ90081	Red algae totivirus 1	30	Fungal virus
THR-E_DN3125	1,465	AWV67014	Lysoka partiti-like virus	47	Fungal virus
THR-E_DN7093	1,673	AWV67012	Lysoka partiti-like virus	65	Fungal virus
THR-E_DN23526	2,468	AKN79252	Alternaria brassicicola mitovirus	91*	Fungal virus
THR-E_DN14430	1,118	YP_009182158	Pleospora typhicola fusarivirus 1	86	Fungal virus
THR-E_DN15320	2,534	QGY72561	Plasmopara viticola lesion associated ourmia-like virus 31	99*	Fungal virus
THR-E_DN23378	2,355	QIR30262	Plasmopara viticola lesion associated mitovirus 39	99*	Fungal virus
THR-B_DN23683	2,827	QIR30272	Plasmopara viticola lesion associated mitovirus 49	97*	Fungal virus
THR-B_DN27856	2,905	QMP82309	Hemipteran rhabdo-related virus OKIAV26	42	Viral insertion
THR-D_DN18510	1,167	QDZ71189	Megastigmus ssRNA virus	29	Viral insertion
THR-E_DN22039	2,499	QMP82403	Hemipteran orthomyxo-related virus OKIAV188	55	Viral insertion
THR-E_DN24098	10,345	QQN90111	Soybean thrips iflavirus 2	56	Viral insertion

a Columns show contig identifier code, contig length (in bases), first viral hit accession number in NCBI:protein, first viral hit organism (NCBI BLASTx first hit), NCBI hit identity, and the origin of the viral contig.

b Asterisks indicate all the blast hits with identity higher than 90.

**TABLE 2:** List of insect-associated viral contigs discovered applying our bioinformatic pipeline to RNA sequencing data of thrips samples^*a*^

Contig	Length (no. of bases)	Accession no.	NCBI BLASTx first hit^*b*^	Identity^*c*^	GenBank accession no.	Suggested virus name	Paper ID	Origin
THR-D_DN15333	5,570	ALV85426	Diaphorina citri densovirus	32	MN764146	Frankliniella occidentalis associated densovirus 1	Foadenso1	Thrips virus
THR-C_DN26815	19,679	BAM99927	Gentian Kobu-sho-associated virus	37	MN714664	Frankliniella occidentalis associated flavi-like virus 1	Foaflavi1	Thrips virus
THR-A_DN28578	9,971	QQN90111	Soybean thrips iflavirus 2**	65	MN714670	Frankliniella occidentalis associated iflavirus 1	Foaifla1	Thrips virus
THR-D_Contig1	19,113	QPD01782	Aphis citricidus meson-like virus	33	MN714663	Frankliniella occidentalis associated mesonivirus 1	Foameso1	Thrips virus
THR-D_DN19783	14,297	QLL27736	Leveillula taurica associated rhabdo-like virus 1	30	MN714688	Frankliniella occidentalis associated mononegavirales virus 1	Foamono1	Thrips virus
THR-A_DN24521	12,116	QPZ88392	Soybean thrips rhabdo-like virus 3**	40	MN714690	Frankliniella occidentalis associated mononegavirales virus 3	Foamono3	Thrips virus
THR-A_DN23655	4,933	YP_009246486	Yogue virus	37	MN764155	Frankliniella occidentalis associated nairo-like virus 1	Foanairo1	Thrips virus
THR-C_DN25036	14,196	YP_009342435	Wuhan house centipede virus 1	25	MN714666	Frankliniella occidentalis associated negev-like virus 1	Foanegev1	Thrips virus
THR-A_DN26080	14,242	AMO03221	Buckhurst virus	24	MN714665	Frankliniella occidentalis associated negev-like virus 2	Foanegev2	Thrips virus
THR-D_DN16267	8,294	YP_009342435	Wuhan house centipede virus 1	25	MN714671	Frankliniella occidentalis associated negev-like virus 3	Foanegev3	Thrips virus
THR-D_DN17549	9,618	QQP18740	Soybean thrips bunya-like virus 1**	31	MN764150	Frankliniella occidentalis associated peribunyavirus-like virus 2 segm1	Foaperi2_Seg1	Thrips virus
THR-D_DN17549	4,566	BBD75426	Ixodes scapularis bunyavirus	28	MN764149	Frankliniella occidentalis associated peribunyavirus-like virus 2 segm2	Foaperi2_Seg2	Thrips virus
THR-D_DN15448	5,780	YP_009342465	Wuhan insect virus 15	28	MN764147	Frankliniella occidentalis associated qin-like virus1 RNA1	Foaqin1_RNA1	Thrips virus
THR-D_DN15448	2,630	YP_009342457	Wuhan insect virus 15	27	MN764148	Frankliniella occidentalis associated qin-like virus1 RNA2	Foaqin1_RNA2	Thrips virus
THR-A_DN15734	2,856	QHA33888	Atrato Sobemo-like virus 2	51	MN725049	Frankliniella occidentalis associated sobemo-like virus 1 RNA1	Foasobemo1_RNA1	Thrips virus
THR-A_DN15734	1,604	AWY11068	Motts Mill virus	38	MN725050	Frankliniella occidentalis associated sobemo-like virus 1 RNA2	Foasobemo1_RNA2	Thrips virus
THR-D_DN18360	12,138	YP_009337819	Hubei virga-like virus 12	38	MN714667	Frankliniella occidentalis associated virga-like virus 2	Foavirga2	Thrips virus
T-Ame_DN13171	3,410	QPZ88398	Soybean thrips sobemo-like virus 4**	99*	MN714676	Neohydatothrips associated sobemo-like virus 1 RNA1	Ntasobemo1_RNA1	Thrips virus
T-Ame_DN13171	1,555	YP_009337912	Hubei diptera virus 13	42	MN714681	Neohydatothrips associated sobemo-like virus 1 RNA2	Ntasobemo1_RNA2	Thrips virus
THR-E_DN17816	7,251	QQM16265	Degsystermes virus	37	MN764152	Thrips tabaci associated bunyavirales 1 RNA1	Ttabunya1_RNA1	Thrips virus
THR-E_DN17816	1,222	AOY18790	Bunyavirus sp.	26	MN764153	Thrips tabaci associated bunyavirales 1 RNA2	Ttabunya1_RNA2	Thrips virus
THR-E_DN23749	10,255	APG79357	Xingshan nematode virus 3	40	MN764156	Thrips tabaci associated bunya-like virus 2	Ttabunya2	Thrips virus
THR-E_DN23726	4,940	APG79254	Shayang ascaridia galli virus 1	38	MN764154	Thrips tabaci associated bunya-like virus 3	Ttabunya3	Thrips virus
THR-E_DN24108	4,897	QIM73965	Riboviria sp.	36	MN764151	Thrips tabaci associated bunya-like virus 4	Ttabunya4	Thrips virus
THR-E_DN24112	12,836	APG78763	Hubei dimarhabdovirus virus 4	73	MN714687	Thrips tabaci associated dimarhabdovirus 1	Ttadima1	Thrips virus
THR-E_DN24095	7,929	QQP18723	Soybean thrips-associated dsRNA virus-1**	39	MN764138	Thrips tabaci associated dsRNA virus 1	Ttads1	Thrips virus
THR-E_DN23955	8,740	QQP18723	Soybean thrips-associated dsRNA virus-1**	56	MN764139	Thrips tabaci associated dsRNA virus 2	Ttads2	Thrips virus
THR-E_DN19401	3,243	QPZ88376	Soybean thrips virus 1**	40	MN764158	Thrips tabaci associated jingmen-like virus 1 RNA1	Ttajing1_RNA1	Thrips virus
THR-E_DN19401	3,089	QPZ88383	Soybean thrips virus 2**	36	MN764159	Thrips tabaci associated jingmen-like virus 1 RNA2	Ttajing1_RNA2	Thrips virus
THR-E_DN7390	3,135	QQM16241	Wifsystermes virus	38	MN714677	Thrips tabaci associated luteo-like virus 1 RNA1	Ttaluteo1_RNA1	Thrips virus
THR-E_DN7390	1,541	AKH40292	Motts Mill virus	40	MN714682	Thrips tabaci associated luteo-like virus 1 RNA2	Ttaluteo1_RNA2	Thrips virus
THR-E_DN24087	1,803	DAD49837	Lutzomyia longipalpis mitovirus 1	41	MN714680	Thrips tabaci associated mitovirus 1	Ttamito1	Thrips virus
THR-B_DN29294	2,816	QDH89080	*Mitovirus* sp.	57	MN714678	Thrips tabaci associated mitovirus 2	Ttamito2	Thrips virus
THR-B_DN29604	3,063	AWY10987	Sclerotinia sclerotiorum mitovirus 29	63	MN714686	Thrips tabaci associated mitovirus 3	Ttamito3	Thrips virus
THR-E_DN24029	1,156	QED42987	Entomophthora narnavirus C	47	MN714683	Thrips tabaci associated narna-like virus 2	Ttanarna2	Thrips virus
THR-E_DN18567	4,450	Q02119	Rice dwarf virus (isolate Akita)	29	MN764140	Thrips tabaci associated reovirus 1 RNA1	Ttareo1_RNA1	Thrips virus
THR-E_DN18567	4,049	NP_620541	Rice ragged stunt virus	23	MN764141	Thrips tabaci associated reovirus 1 RNA2	Ttareo1_RNA2	Thrips virus
THR-E_DN18567	3,679	QIJ56929	Scaphoideus titanus reo-like virus 1	31	MN764142	Thrips tabaci associated reovirus 1 RNA3	Ttareo1_RNA3	Thrips virus
THR-E_DN18567	3,207	QHA33826	Atrato reo-like virus	25	MN764143	Thrips tabaci associated reovirus 1 RNA4	Ttareo1_RNA4	Thrips virus
THR-E_DN18567	2,522	QIJ56928	Scaphoideus titanus reo-like virus 1	31	MN764144	Thrips tabaci associated reovirus 1 RNA5	Ttareo1_RNA5	Thrips virus
THR-B_DN22880	4,173	QQP18784	Soybean thrips tombus-like virus 3	53	MN714675	Thrips tabaci associated tombus-like virus 1	Ttatombus1	Thrips virus
THR-B_DN23975	6,965	YP_009337819	Hubei virga-like virus 12	39	MN714672	Thrips tabaci associated virga-like virus 1 RNA1	Ttavirga1_RNA1	Thrips virus
THR-B_DN23975	3,565	YP_009336553	Hubei virga-like virus 9	26	MW459159	Thrips tabaci associated virga-like virus 1 RNA2	Ttavirga1_RNA2	Thrips virus
THR-E_DN21965	5,712	YP_009337854	Shahe yuevirus-like virus 1	25	MN764157	Thrips tabaci associated yue-like virus 1 RNA1	Ttayue1_RNA1	Thrips virus
THR-E_DN21965	1,853		No significant similarity found	0	MW297844	Thrips tabaci associated yue-like virus 1 RNA2	Ttayue1_RNA2	Thrips virus
THR-E_DN23836	18,939	YP_009026378	Casuarina virus	28	MN714662	Insect metagenomics mesonivirus 1	Immeso1	Insect virus
THR-D_DN18103	11,139	YP_009505536	Piry virus	41	MN714689	Insect metagenomics mononegavirales virus 2	Immono2	Insect virus
THR-D_DN17285	3,058	QNQ74064	Plasmopara viticola lesion associated orfanplasmovirus 2	29	MN764145	Insect metagenomics narna-like virus 1 RNA1	Imnarna1_RNA1	Insect virus
THR-D_DN17285	2,461	YP_001497520	Paramecium bursaria Chlorella virus NY2A	43	MW297845	Insect metagenomics narna-like virus 1 RNA2	Imnarna1_RNA2	Insect virus
THR-E_DN23507	2,646	YP_009333179	Beihai barnacle virus 10	42	MN714679	Insect metagenomics narna-like virus 2	Imnarna2	Insect virus
THR-E_DN1552	2,411	QMP82343	Hemipteran orthomyxo-related virus OKIAV188	32	MN764160	Insect metagenomics orthomyxo-like virus 1 RNA1	Imortho1_RNA1	Insect virus
THR-E_DN1552	2,263	QMP82299	Hemipteran orthomyxo-related virus OKIAV188	42	MN764161	Insect metagenomics orthomyxo-like virus 1 RNA2	Imortho1_RNA2	Insect virus
THR-E_DN1552	1,733	QMP82317	Hemipteran orthomyxo-related virus OKIAV188	34	MN787040	Insect metagenomics orthomyxo-like virus 1 RNA3	Imortho1_RNA3	Insect virus
THR-E_DN1552	1,075	AAN34384	Human immunodeficiency virus 1	31	MW297846	Insect metagenomics orthomyxo-like virus 1 RNA4	Imortho1_RNA4	Insect virus
THR-E_DN23568	9,979	QKN89031	Riboviria sp.	35	MN714669	Insect metagenomics picorna-like virus 1	Impico1	Insect virus
THR-A_DN18812	3,345	YP_009330120	Hubei sobemo-like virus 25	76	MN725051	Insect metagenomics sobemo-like virus 2 RNA1	Imsobemo2_RNA1	Insect virus
THR-A_DN18812	1,549	AKP18619	Humaita-Tubiacanga virus	46	MN725052	Insect metagenomics sobemo-like virus 2 RNA2	Imsobemo2_RNA2	Insect virus
THR-A_DN28235	3,634	AXA52557	Linepithema humile C virus 1	53	MN787041	Insect metagenomics tombusbipa-like virus 1 RNA1	Imtombus2_RNA1	Insect virus
THR-A_DN28235	2,271	AXA52559	Linepithema humile C virus 1	33	MN787042	Insect metagenomics tombusbipa-like virus 1 RNA2	Imtombus2_RNA2	Insect virus
THR-D_DN14008	6,340	QDZ71189	Megastigmus ssRNA virus	35	MN714674	Insect metagenomics virga-like virus 1 RNA1	Imvirga1_RNA1	Insect virus
THR-D_DN14008	2,038	YP_009337242	Hubei virga-like virus 11	48	MW459158	Insect metagenomics virga-like virus 1 RNA2	Imvirga1_RNA2	Insect virus

a Columns show contig identifier code, contig length (in bases), first viral hit accession in NCBI:protein, first viral hit organism (NCBI BLASTx first hit), NCBI hit identity, new discovered virus identifier (ID) (GenBank accession no.), suggested virus name, the virus abbreviation (ID) in the paper, and the origin of the viral contig.

b Double asterisks indicate all the hits from reference 16.

c Asterisks indicate all the blast hits with identity higher than 90.

**FIG 3:**
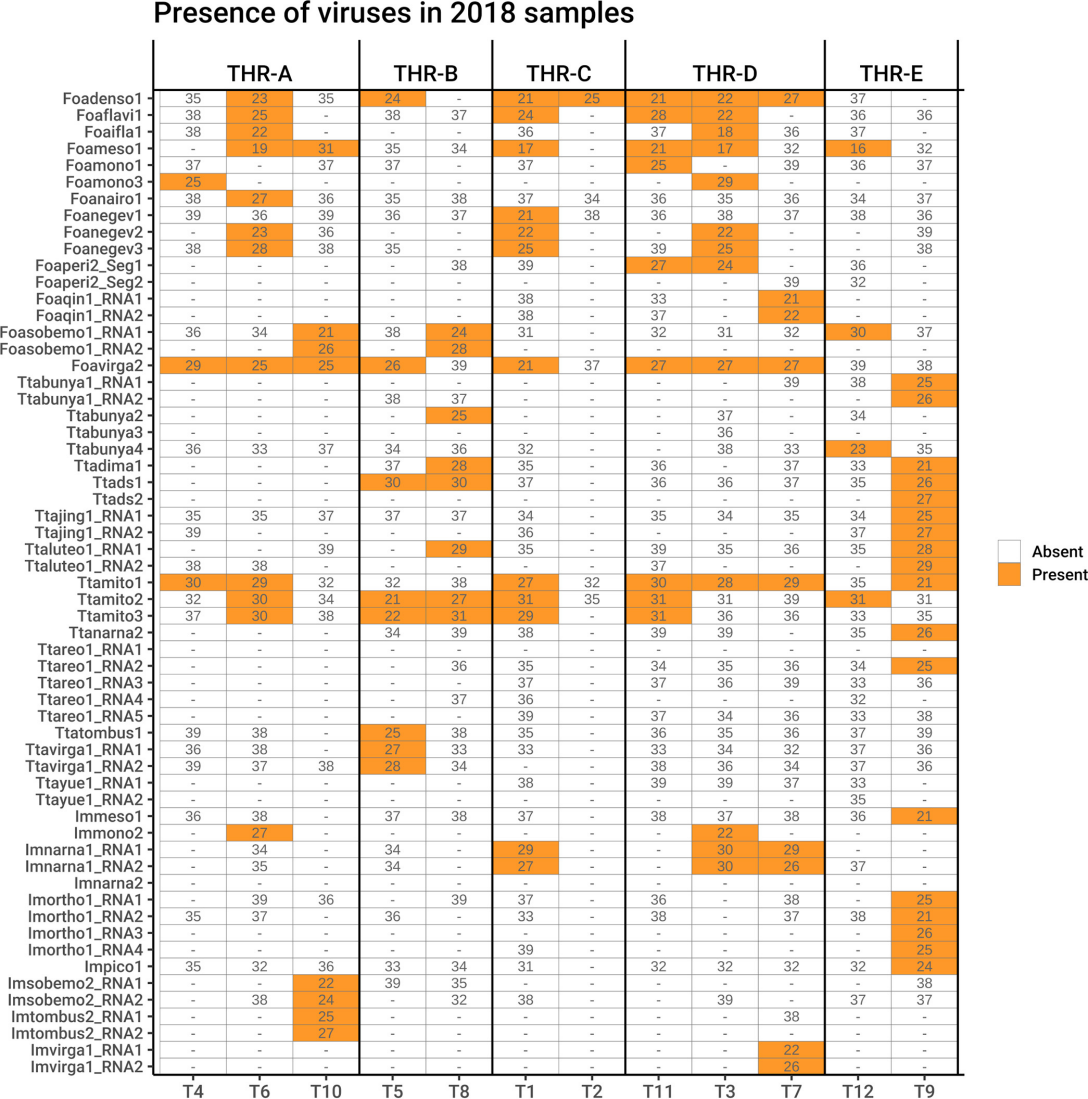
Graphical representation of qRT-PCR results (expressed as *C_T_* number) showing the presence of each viral contig in each RNA sample. Above the heat map the library names are indicated, whereas below the table are the individual samples of the libraries.

Each virus contig was tracked to each sample by real-time reverse transcription-quantitative PCR (qRT-PCR), and threshold cycle (*C_T_*) values were used as an approximate indication of relative accumulation of viral RNA; *C_T_* values above 31 were not considered reliable, and so samples with amplifications after that cycle were considered negative (Fig. 3).

**FIG 4:**
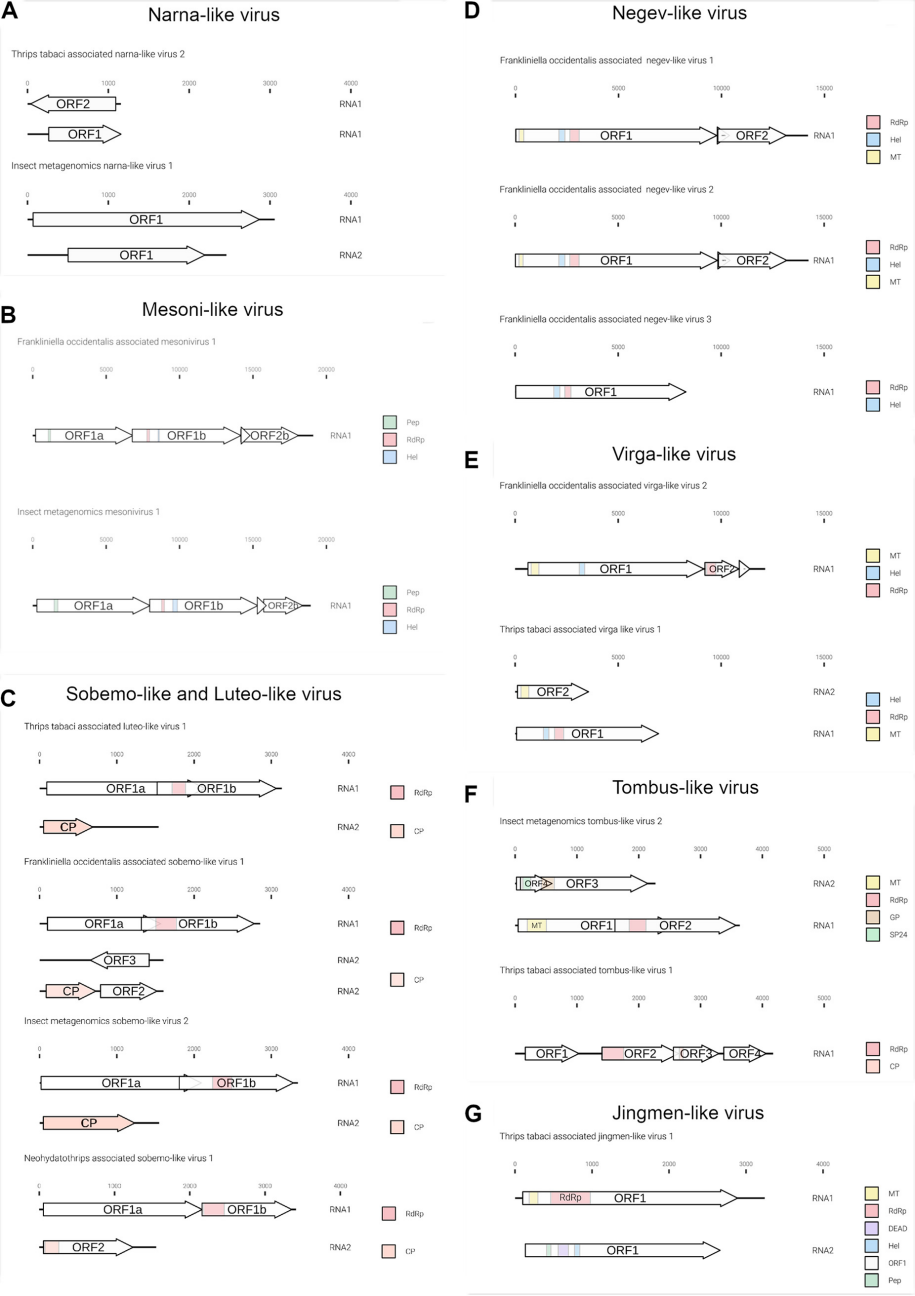
Graphical representation of the novel genome organizations of plus-strand RNA viruses associated with *Frankliniella occidentalis* and *Thrips tabaci* viromes. (A) New narna-like viruses; (B) new mesoni-like viruses; (C) Sobemo/luteo-like viruses; (D) new negev-like viruses; (E) new virga-like viruses; (F) new tombus-like viruses; (G) jingmen-like viruses. RdRP, RNA-dependent RNA polymerase; Pep, peptidase; Hel, helicase; CP, coat protein; MT, methyltransferase; GP, glycoprotein; SP24, putative virion membrane protein of plant and insect virus; DEAD, DEAD box helicases. Arrows represent open reading frames (ORFs).

**FIG 5:**
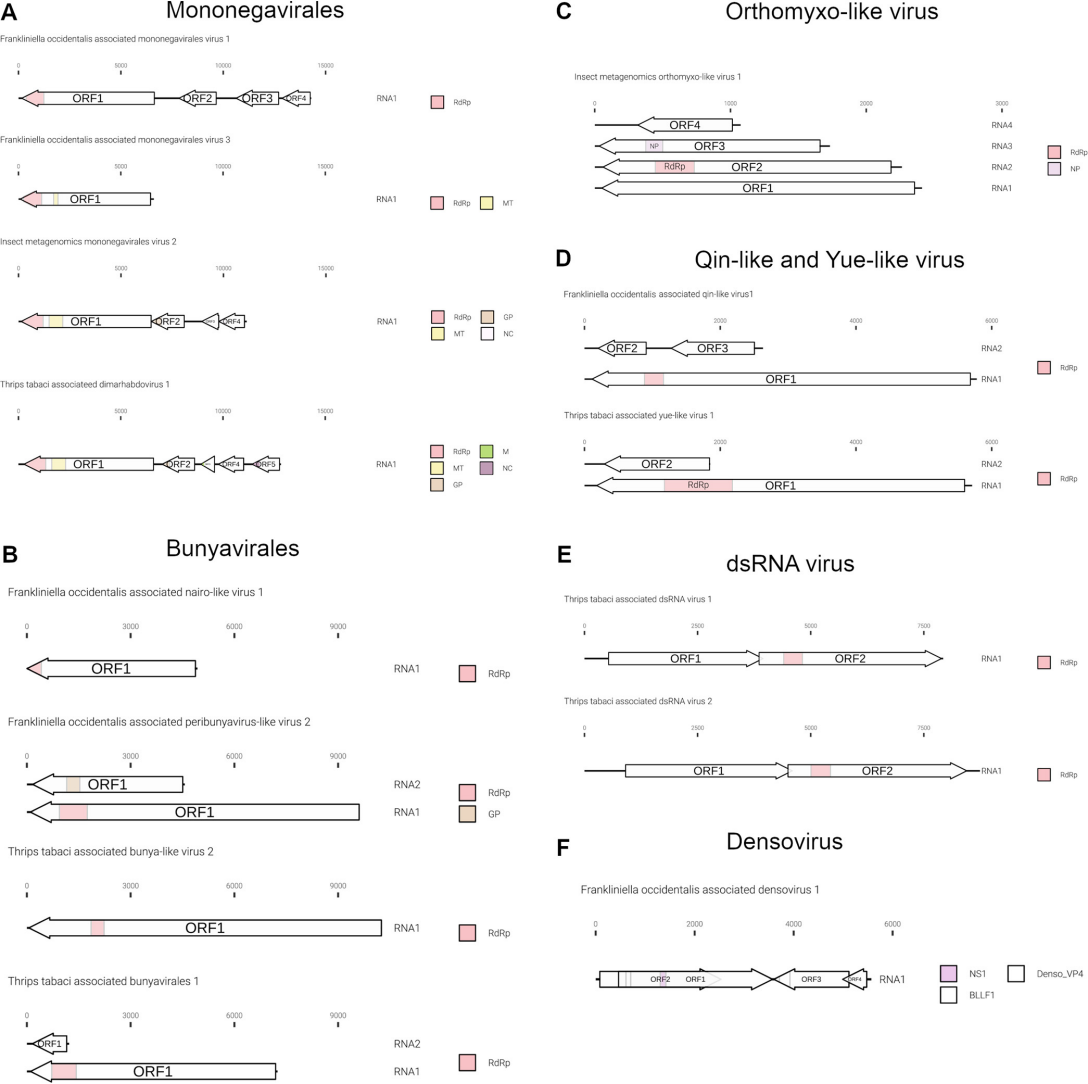
Graphical representation of the novel genome organizations of negative-strand, dsRNA virus and a densovirus-like sequence characterized from *Frankliniella occidentalis* and *Thrips tabaci* viromes. (A) Genomes in order *Mononegavirales*; (B) genomes in order *Bunyavirales*; (C) genome organization of an orthomyxo-like virus; (D) yue-like and qin-like virus genome organizations; (E) genome organization of two dsRNA viruses; (F) densovirus genome organization. MP, matrix protein domain; NS1, nonstructural protein 1; Denso_VP4, capsid protein VP4; BLLF1, envelope glycoprotein GP350. Arrows represent open reading frames.

The genome organizations of the most abundant and previously undescribed viral contigs are displayed in Fig. 4 and 5.

### (i) Phylum *Lenarnaviricota*.

In our study, we identified three mitoviruses from *T. tabaci*, named Thrips tabaci associated mitoviruses 1, 2, and 3 (Ttamito1, Ttamito2, and Ttamito3) (Table 2). Based on read counts (Fig. 2), Ttamito1 is abundant in THR-E, and this observation is also confirmed by qRT-PCR results (Fig. 3), even if the qRT-PCR also detected the low-abundance presence of Ttamito1 in T4 to T6 (THR-A), T1 (THR-C), and T3, T7, and T11 (THR-D). Ttamito2 and Ttamito3 are more abundant in THR-B (Fig. 2) although present also in THR-A, THR-D, and THR-E. The qRT-PCRs confirmed the read count results, showing the same accumulation pattern (Fig. 3).

Three narnaviruses have also been identified: one from *T. tabaci*, named Thrips tabaci associated narnavirus 2 (Ttanarna2), and the remaining two from unconfirmed origin named insect metagenomics narnavirus 1 (Imnarna1) and insect metagenomics narnavirus 2 (Imnarna2), respectively (Table 2). The latter is present in low abundance in both THR-B and THR-E based on read counts, but we were not able to amplify it in qRT-PCR. Imanarna1, instead, is bipartite and mostly concentrated in THR-C and THR-D, based on read counts and qRT-PCR (Fig. 2 and 3). The second genomic RNA segment of Imanarna1 was detected not from BLAST similarity searches but from ORFans analysis linked to correlation in mapped-read abundance in different libraries and specific sample infection.

**FIG 6:**
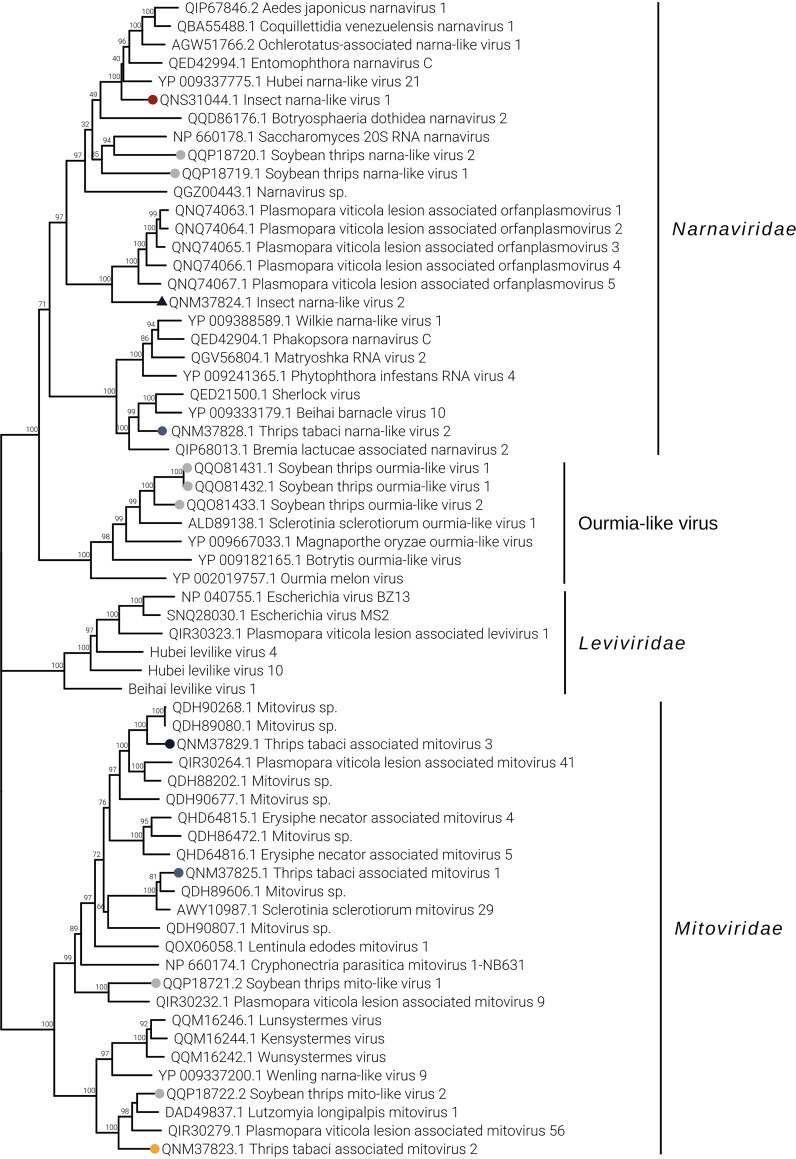
Lenarnaviricota phylogenetic tree computed by IQ-TREE stochastic algorithm to infer phylogenetic trees by maximum likelihood. The model of substitution is VT+F+I+G4. The consensus tree is constructed from 1,000 bootstrap trees. The log likelihood of the consensus tree is −113395.268. At nodes are the percent bootstrap values. Different colors indicate different subgroups. Triangles indicate the insect metagenomics viruses, and circles indicate *Thrips tabaci*- or *Frankliniella occidentalis*-associated viruses. Viruses labeled with a gray circle are the ones identified in a recent soybean thrip virome characterization study (16).

Genomic organization of mitoviruses show a monopartite genome, and all three have an RNA-dependent RNA polymerase (RdRP) domain (data not shown). While Ttamito2 and Ttamito3 have a genome size around 3 kb, Ttamito1 displays a genome shorter than 2 kb, with a complete open reading frame (ORF). On the other hand, none of the three narnaviruses identified in this study presents a conserved RdRP domain comparable to those already present in the databases, even if the BLAST search of the ORF1 of each virus returns as a first hit an RdRP of another virus. Sequence alignment shows that indeed the most conserved palm subdomains are partly conserved, including the GDD motif (data not shown). Moreover, Imnarna2 does not carry a complete ORF1, probably limited coverage did not allow to recover the complete genomic sequence. Interestingly, Ttanarna2 shows a very short genome and a putative ambisense ORF organization (Fig. 4A). Based on our phylogenetic analysis (Fig. 6), this virus lies in the same clade as Saccharomyces 20S RNA narnavirus (the reference virus for the *Narnavirus* genus). Imnarna2 clusters with the recently characterized orfanplasmoviruses (17), but in more basal position, while Imnarna1 does not. Mitoviruses are in a separate clade from the narnavirus, but while Ttamito1 falls in a clade that includes a putative insect virus (Wenling narna-like virus 9), Ttamito2 and Ttamito3 fall in clades with conserved fungal mitoviruses. Overall, the three mitoviruses we here describe are each in a distinct clade, possibly different viral genera.

### (ii) Phylum *Pisuviricota*.

Two virus contigs had as first hit a member of the *Mesoniviridae* family (Table 2) using BLASTx to search the databases. Mesoniviruses are viruses with a genome size around 20 kb, which is intermediate among the size ranges of nidoviruses. Typical mesoniviruses are monoparite, with a linear genome containing 7 ORFs (ORF1a, ORF1b, ORF2a, ORF2b, ORF3a, ORF3b, and ORF4), encoding an RdRP protein and other nonstructural (NS) proteins involved in RNA synthesis and at least two structural proteins. The primary identified hosts for mesoniviruses were insects; in fact, this family represents the first nidoviruses to be discovered in insects (18).

**FIG 7:**
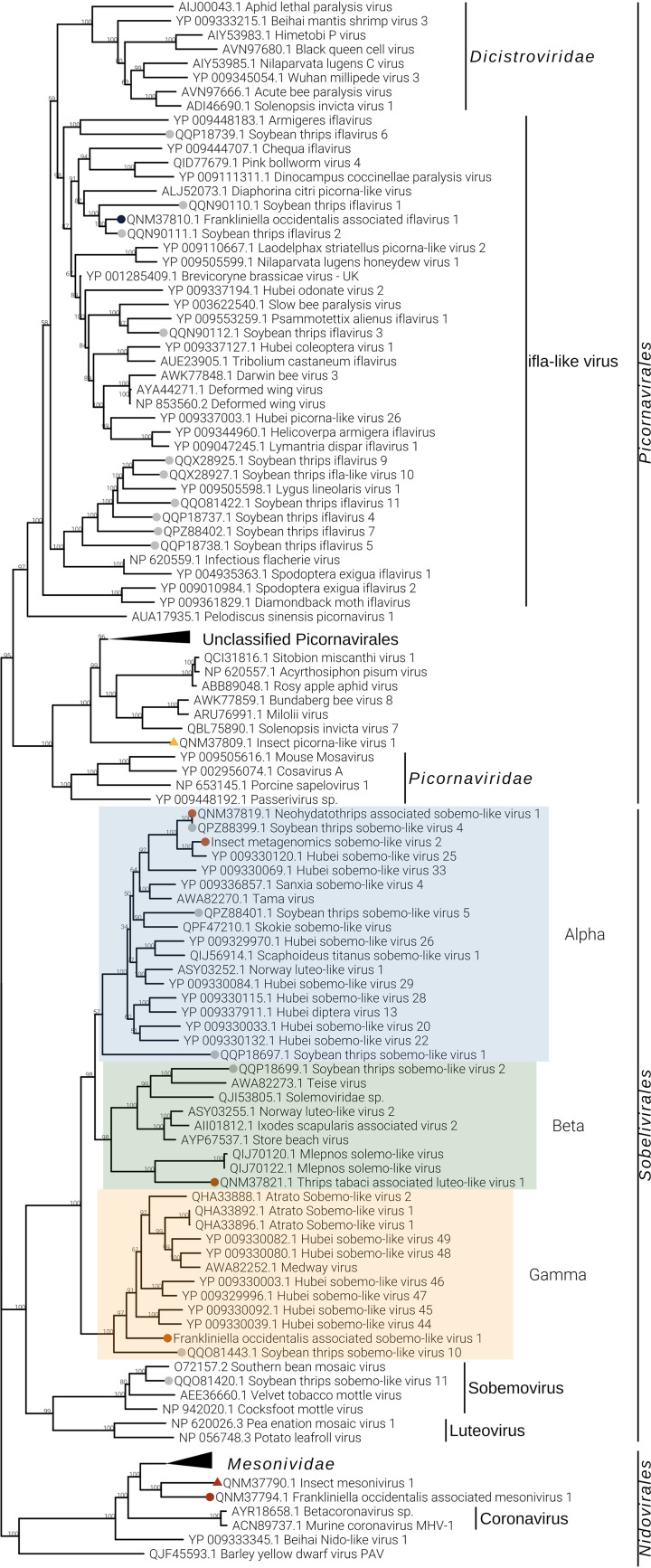
*Pisuviricota* phylogenetic tree computed by IQ-TREE stochastic algorithm to infer phylogenetic trees by maximum likelihood. The model of substitution is VT+F+I+G4. The consensus tree is constructed from 1,000 bootstrap trees. The log likelihood of the consensus tree is −504786.937. At nodes are the percent bootstrap values. Different colors indicate different subgroups. Triangles indicate the insect metagenomics viruses, and circles indicate *Thrips tabaci*- or *Frankliniella occidentalis*-associated viruses. Viruses labeled with a gray circle are the ones identified in a recent soybean thrip virome characterization study (16).

The two mesoni-like viruses identified in our study were named insect metagenomics mesonivirus 1 (Immeso1) and Frankliniella occidentalis associated mesonivirus 1 (Foameso1), both slightly shorter than 20 kb. Immeso1, 19 kb (GenBank accession no. MN714662), was found in only one pool (THR-E) from the north of Italy, while Foameso1, 19 kb (GenBank accession no. MN714663), was found in three different pools (THR-A, THR-C, and THR-D). Based on read counts (Fig. 2) and qRT-PCR (Fig. 3), we can infer that these mesoniviruses are highly concentrated in the samples. A phylogenetic tree (Fig. 7) derived from alignment of ORF1b (containing the RdRP) shows Immeso1 and Foameso1 clustering together in a well-supported branch (bootstrap value, 97) separated from the *Mesoniviridae* family clade, indicating these two viruses can be a new genus inside the *Mesoniviridae* family or possibly a new family in the order *Nidovirales*.

The genomic organizations of the two mesoniviruses are identical: both present four ORFs. ORF 2a and ORF2b do not carry any conserved domain present in the databases but are conserved, respectively, with the putative mesonivirus spike protein (S) and nucleocapsid protein (N). ORF1a has a peptidase domain and ORF1b contains the helicase (Hel) and RdRP domains (Fig. 4B). Typical mesoniviruses so far characterized from insects include 2 further ORFs (ORF3a and −3b) and often, but not always, a 4th ORF (18).

Two new sobemo-like viruses and one luteo-like virus have been discovered and named Foasobemo1, Imsobemo2, and Ttaluteo1, respectively. One sobemo-like virus was also identified in the sample from the United States and named Neohydatothrips associated sobemo-like virus 1 (Ntasobemo1). Read counts (Fig. 2) and qRT-PCR (Fig. 3) show the presence of Ttaluteo1 virus in library THR-E and, in particular, in sample T9; its presence in library THR-B seems to be strongly supported by the read counts. Foasobemo1 and Imsobemo2 are present in two pools out of five: THR-A and THR-B. Specifically, Foasobemo1 is present in samples T10 (THR-A) and T8 (THR-B) based on qRT-PCR results (Fig. 3), while Imsobemo2 is only amplified in T10 (THR-B) (Fig. 3). Both segments of Ntasobemo1 were present only in T-Ame sample at very high concentration (Fig. 3).

Phylogenetic analysis (Fig. 7) clearly shows that the three new Italian viruses from thrips are in distinct clades each defining a new viral family, while Ntasobemo1 clusters in a sister clade of Imsobemo2. These new viruses do not belong to either the *Sobemovirus* or *Luteovirus* genus. The phylogenetic tree reports three taxonomically classified sobemoviruses (cocksfoot mottle virus [NCBI:protein accession no. NP_942020.1], velvet tobacco mottle virus [AEE36660.1], and southern bean mosaic virus [O72157.2]) and three luteoviruses (barley yellow dwarf virus PAV [QJF45593.1], pea enation mosaic virus 1 [NP_620026.3], and potato leafroll virus [NP_056748.3]), and they form a distinct clade from the putative thrips-associated viruses we report. Foasobemo1, Imsobemo2, and Ttaluteo1 cluster in the same parent clade but form three distinct putative new families very well supported by the bootstrap values, labeled Alpha, Beta, and Gamma on the tree.

Viruses belonging to the *Sobemovirus* and *Luteovirus* genera are monopartite plant viruses with genomes of circa 4 kb and 5 kb, respectively. The four viruses we identified (Foasobemo1, Imsobemo2, Ttaluteo1, and Ntasobemo1) are instead bipartite insect viruses. Viruses present on the tree, like Scaphoideus titanus sobemo-like virus 1 (19) for clade Alpha, Wuhan heteropteran virus 2 (14) for clade Beta, and Hubei sobemo-like virus 47 (14) for clade Gamma, are also insect viruses with bipartite genomes, so official recognition and new names for these new taxa are needed.

Ttaluteo1 and Imsobemo2 show similar genomic organizations (Fig. 4C): RNA1 (∼3.2 kb) contains two ORFs translated via a −1 ribosomal frameshift, and one of them contains the RdRP motif (ORF1b). RNA2 (∼1.5 kb) contains the putative capsid protein. Foasobemo1 displays the same genomic organization for RNA1, while RNA2 shows the presence of the putative capsid protein plus two additional ORFs: ORF2 (∼0.7 kb) in the sense direction and ORF3 (∼0.7 kb) in the antisense direction. Neither of the two ORFs show conserved domains.

One ifla-like virus has been identified from *F. occidentalis* and named Foaifla1 (Frankliniella occidentalis associated ifla-like virus 1). The ifla-like virus shows a monopartite genome of about 10 kb, coding for a polyprotein with multiple domains. In the phylogenetic tree (Fig. 7), the taxonomically accepted iflavirus do not cluster with Foaifla1, but Foaifla1 shares a branch with another putative thrips virus from *N. variabilis* (16), supporting the contention that these are both indeed thrips viruses. The clade containing our identified virus also contains a mix of iflavirus and picornaviruses, which probably need to be accommodated in a new genus. Based on read counts and qRT-PCR, Foaifla1 is tracked to 3 pools (THR-A [T6], THR-C, and THR-D [T3]) (Fig. 2 and 3), but the qRT-PCR results only partially confirm the read count evidence: Foaifla1 was not amplified in THR-C library despite the fact that the reads count showed a high concentration, raising the possibility that a specific variant of this virus in the region where primers anneal makes this virus not detectable.

One picorna-like virus has been identified in THR-E pool (northwest of Italy), sample T9, named insect metagenomics picorna-like virus 1 (Impico1) (Fig. 3). Impico1 has a genome of ∼10 kb, slightly longer than a taxonomically characterized picornavirus but aligned with insect picorna-like viruses (14). The monopartite genome is similar to that of picorna-like viruses (Fig. 5) but shows an additional ORF with an arginine/serine-rich protein PNISR domain (PF15996). ORF1 contains two domains, i.e., a helicase domain and an RdRP domain, while ORF2 contains the coat protein domain. Phylogenetic analysis shows taxonomically accepted *Picornaviridae* (cosavirus A, mouse mosavirus, porcine sapelovirus 1, and *Passerivirus* sp.) not in the same clade as our newly identified virus (Fig. 7). So, based on genomic organization and phylogenetic tree topology, Impico1 virus requires the establishment of a new family to accommodate it.

### (iii) Phylum *Kitrinoviricota*.

Frankliniella occidentalis associated flavi-like virus 1 (Foaflavi1) has been identified in all three pools containing *F. occidentalis*, always well represented by number of reads mapping the genome, especially in sample THR-C, where the read count is over 29,000 (Fig. 2). Real time RT-PCRs confirmed the presence of Foaflavi1 in all the above-mentioned pools (Fig. 3). Compared to typical *Flavivirus*, usually with a genome of about 10 to 11 kb, Foaflavi1 has a double-size genome, almost 20 kb, a feature shared with some recently characterized insect-specific flaviviruses identified in Apis mellifera L. and Diaphorina citri Kuwayama (20, 21). The genomic organization shows a monopartite, linear single-stranded RNA positive-sense [ssRNA(+)] genome, with a single ORF that produces a putative polyprotein (Fig. 5). MOTIF Search was not able to identify the RdRP signature, but the GDD motif is present and can be aligned to flavi-like virus palm domains (data not shown).

**FIG 8:**
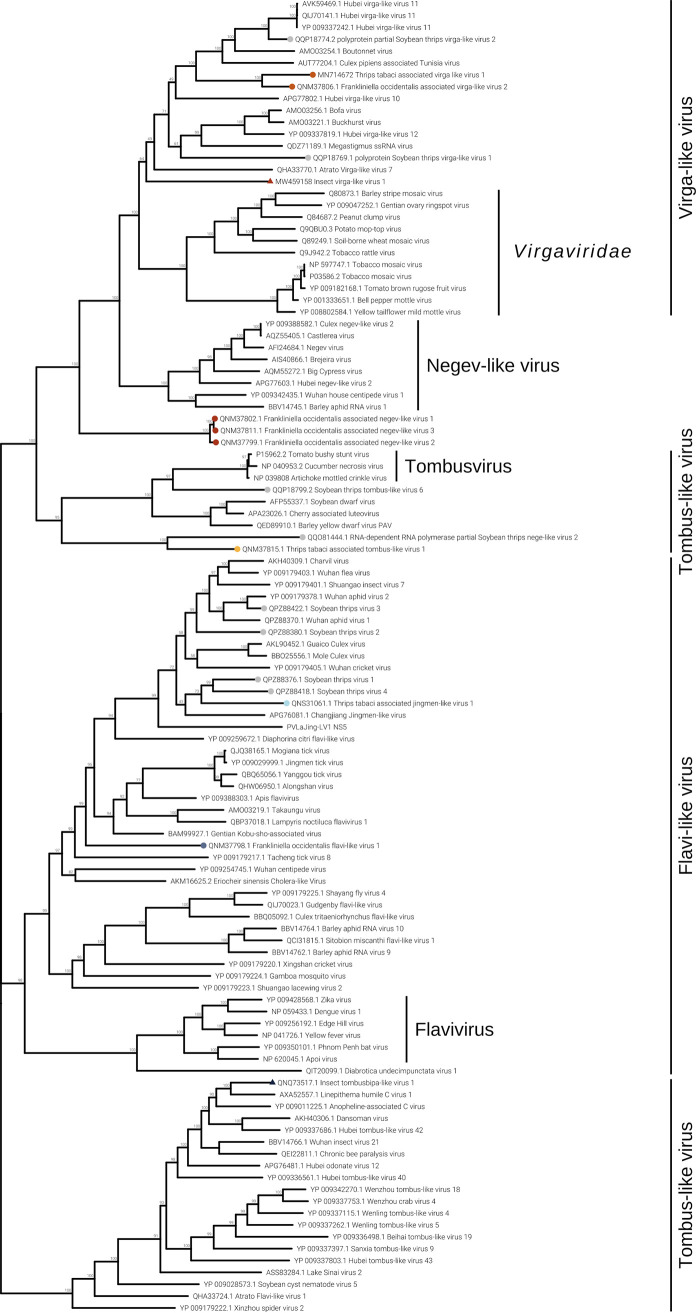
*Kitrinoviricota* phylogenetic tree computed by IQ-TREE stochastic algorithm to infer phylogenetic trees by maximum likelihood. The model of substitution is Blosum62+I+G4. The consensus tree is constructed from 1,000 bootstrap trees. The log likelihood of the consensus tree is −529281.876. At nodes are the percent bootstrap values. Different colors indicate different subgroups. Triangles indicate the insect metagenomics viruses, and circles indicate *Thrips tabaci*- or *Frankliniella occidentalis*-associated viruses. Viruses labeled with a gray circle are the ones identified in a recent soybean thrip virome characterization study (16).

The phylogenetic analysis shows Foaflavi1 in a sister clade of members of the genus *Flavivirus*, with other flavi-like viruses from insects (Fig. 8).

Three negev-like viruses have been identified in *F. occidentalis*: Frankliniella occidentalis associated negev-like virus 1 (Foanegev1), Frankliniella occidentalis associated negev-like virus 2 (Foanegev2), and Frankliniella occidentalis associated negev-like virus 3 (Foanegev3). Each virus has its peak concentration in a different pool (Fig. 2) and was tracked by qRT-PCR to distinct samples: Foanegev1 in THR-C (T1), Foanegev2 in THR-A (T6), and Foanegev3 in THR-D (T3); Foanegev1 is 10 and 30 times more concentrated than Foanegev2 and Foanegev3, respectively (Fig. 2). These three new viruses have been called, temporarily, negev-like viruses, but based on tree topology (Fig. 8), neither of these viruses is a real negevirus. Figure 4D shows that Foanegev1 and Foanegev2 have the same genomic organization and the same domain distribution, while Foanegev3 has a shorter genome, with one single ORF, and the methyltransferase (MT) domain is absent: likely this virus genome is not complete, since it is very similar and phylogenetically related to the other two that are present in the database. Both ORF2 and ORF3 do not have any reliable homologue in NCBInr (virus limited: taxid 10239) in a BLASTp (protein-protein BLAST) search with default parameters.

Three virga-like viruses were identified in our thrips metatranscriptomic analysis, Imvirga1, Foavirga2, and Ttavirga1 (Fig. 2 and Table 2): one putatively from *F. occidentalis* and one putatively from *T. tabaci*; a third one (Imvirga1, a bipartite virus), which could not be assigned to a specific host according to our more stringent criteria, has been identified only in sample T7 (THR-D) (Fig. 3). Conversely, Foavirga2, a monopartite virus, has been identified and amplified in all samples of the THR-A and THR-D libraries, but only in one sample for the libraries THR-B (T5) and THR-C (T1) (Fig. 3).

The virga-like viruses identified from *T. tabaci* displayed in pool THR-B a relative low level of read coverage for both its segments, and qRT-PCR confirmed their presence only in sample T5 (Fig. 2 and 3).

Two out of three virga-like viruses (Imvirga1 and Ttavirga1) show similar genome sizes, about 6.5 kb, for RNA1 and possess a second genomic segment (RNA2) of circa 3.5 and 2.5 kb in length, while Foavirga2 has a longer monopartite genome (∼12 kb). All the identified virgaviruses display 3 domains: RNA-dependent RNA polymerase (RdRP), MT, and Hel, even if their genomic organizations present significant differences. Ttavirga1 has the RdRP and the Hel domains on RNA1 and the MT domain on RNA2. Foavirga2 shows a genomic organization very similar to that of Ttavirga1, but on a single genomic segment, and it includes an ORF that carries the tobamovirus coat protein motif (ORF3) (Fig. 4E).

Our phylogenetic analysis indicates that the viruses we characterized from thrips are clearly only distantly related to the plant family *Virgaviridae* and probably need establishment of a new family taxon to be accommodated (Fig. 8).

Members of the family *Tombusviridae* are worldwide-spread viruses, causing several diseases in plants, but tombus-like viruses are also present in arthropods (14). Thrips tabaci associated tombus-like virus 1 (Ttatombus1) has been identified in THR-B pool (sample T10), while insect metagenomics tombus-like virus 2 (Imtombus2) has been identified in pool THR-A (Fig. 2 and 3). Official members of the family *Tombusviridae* are monopartite viruses with a genome between 4 kb and 5.4 kb. Ttatombus1 has these features but clusters in a sister clade of plant tombusviruses (Fig. 8) and presents a slightly different genomic organization. Tomato bushy stunt virus (reference genome for tombusvirus) has five ORFs. Ttatombus1 (Fig. 4F) has four ORFs, and the RdRP domain is located on ORF2. ORF3 contains the coat protein domain. ORF1 and ORF4 do not contain known motifs based on MOTIF Search analysis and the default BLASTp on the NCBInr database (June 2020) limited to viruses (taxid 10239) did not find significant similarities. Imtombus2 is instead bipartite, and the first match for RNA1 in NCBInr (Oct 2018) is Linepithema humile C virus 1 from Argentine ant (22). Phylogenetic analysis shows the two tombus-like viruses to cluster in different well-separated clades. In particular, Ttatombus1 clusters in a sister group of real tombusvirus, while Imtombus2 clusters with mostly unclassified viruses (Fig. 8). Both viruses need a new family to accommodate them in the current viral taxonomical framework.

Jingmen viruses are segmented positive-sense single-stranded RNA viruses associated with arthropods (23). In our study, we identified one jingmen-like virus in pool THR-E, and we called it Thrips tabaci associated jingmen-like virus 1 (Ttajing1), and the closest hits in the databases are soybean thrips-associated viruses (16). RNA1 and RNA2 have been associated based on read counts (Fig. 2), qRT-PCR results (Fig. 3), and 5′ and 3′ end alignments (data not shown). The viral genomic organization is shown in Fig. 4G; on RNA1 two domains are present, a methyltransferase domain and an RdRP domain, while on RNA2, we identified three domains: a helicase domain, a peptidase domain, and a DEAD domain. A phylogenetic tree (Fig. 8) shows the known jingmen viruses (infecting ticks) clustering in a separate branch from Ttajing1_RNA1 virus. Moreover, Ttajing1_RNA1 virus is basal to the cluster of insect-infecting jingmen-like viruses.

### (iv) Phylum *Negarnaviricota*.

Members of the order *Mononegavirales* have been identified in all five pools: one virus is associated with *T. tabaci* samples (Thrips tabaci associated dimarhabdovirus 1 [Ttadima1]), and two viruses are associated with *F. occidentalis* (Frankliniella occidentalis associated mononegavirales virus 1 and Frankliniella occidentalis associated mononegavirales virus 3 [Foamono1 and Foamono3]) (Fig. 2). One could not be confirmed to be associated with any thrips species according to our strict criteria, so it has been called insect metagenomics mononegavirales virus 2 (Immono2). Based on read counts, three out of four mononegaviruses are not highly concentrated, apart from Ttadima1 in pool THR-E (Fig. 3).

Foamono1 and Immono2 viruses have quite similar genomic organizations (Fig. 5A): they code for four ORFs, and for each of them, the RdRP domain is present in ORF1. While for Foamono1 we were able to identify only the RdRP domain, for Immono2 and Ttadima1 we identified several domains. The Foamono3 genome segment displays only 1 ORF, coding for the putative RdRP and a putative methyltransferase. The Ttadima1 genome has 5 ORFs coding for an RdRP (ORF1), a methyltransferase (ORF1), a glycoprotein (ORF2), a matrix protein (ORF3), and a nucleocapsid (ORF5).

**FIG 9:**
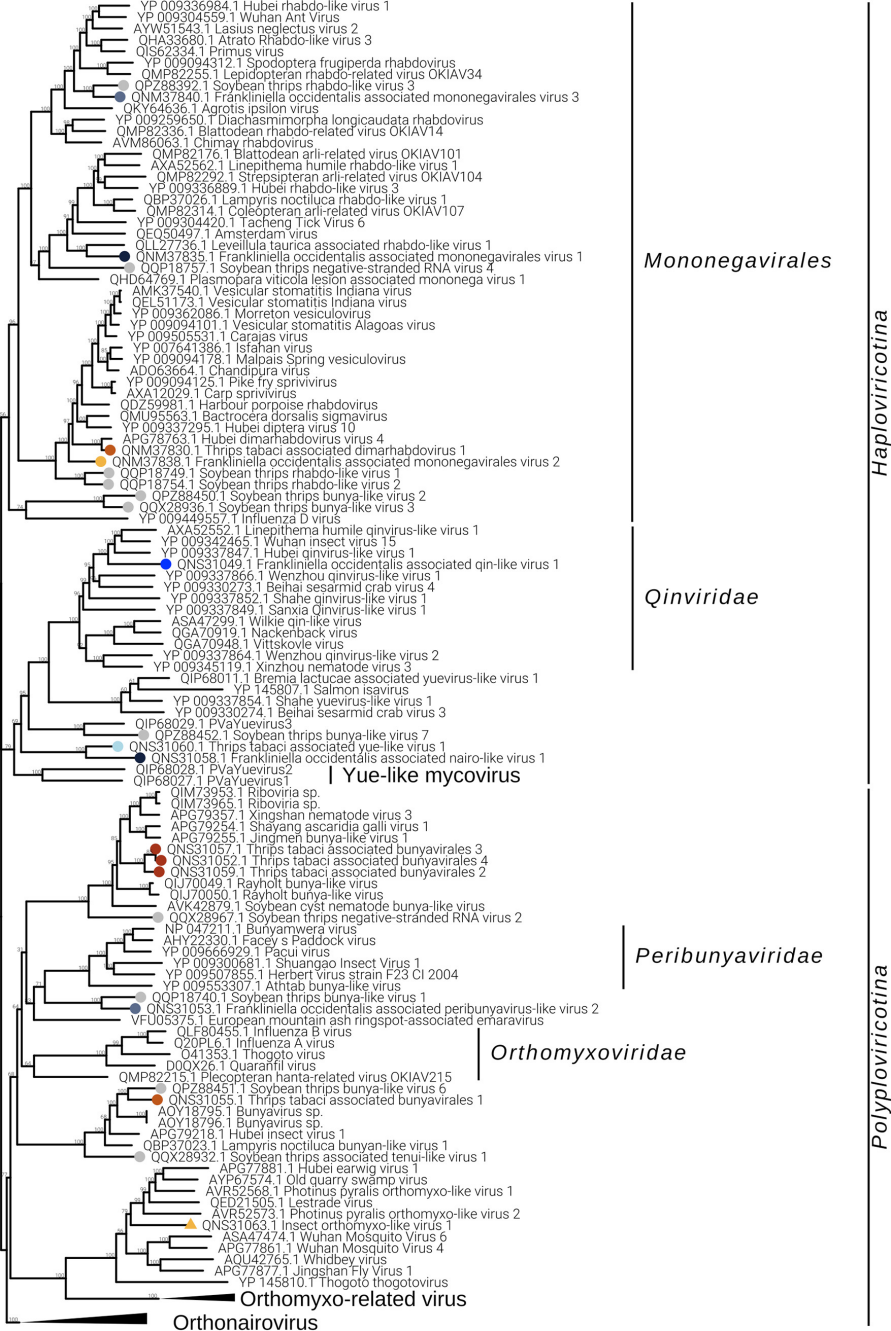
*Negarnaviricota* phylogenetic tree computed by IQ-TREE stochastic algorithm to infer phylogenetic trees by maximum likelihood. The model of substitution is VT+F+G4. The consensus tree is constructed from 1,000 bootstrap trees. The log likelihood of the consensus tree is −526045.530. At nodes are the percent bootstrap values. Different colors indicate different subgroups. Triangles indicate the insect metagenomics viruses, and circles indicate *Thrips tabaci*- or *Frankliniella occidentalis*-associated viruses. Viruses labeled with a gray circle are the ones identified in a recent soybean thrip virome characterization study (16).

Phylogenetic analysis shows that Foamono3 groups with an *N. variabilis* mononegavirus, which has a larger genomic segment that includes at least three extra ORFs (16) (Fig. 9); it is therefore likely that Foamono3 is an incomplete genome. The other three identified members of *Mononegavirales* are in a sister clade of Foamono3 but on different, well-sustained branches, indicating that these viruses could represent different subfamilies/genera.

Members of the order *Bunyavirales* are single-stranded, segmented, linear negative-sense viruses infecting plants, fungi, vertebrates, and invertebrates. The genome size and organization are family dependent. We identified six bunyavirus RdRPs, two in WFT and the remaining four in OT. For Frankliniella occidentalis associated nairovirus 1 (Foanairo1), present only in THR-A, we identified one segment of 4.9 kb; for Frankliniella occidentalis associated peribunyavirus 1 (Foaperi1), present in THR-D, we assembled two segments of 9.6 kb and 4.5 kb (Fig. 5B). We also identified four OT-associated bunyavirus, named Ttabunya1, Ttabunya2, Ttabunya3, and Ttabunya4, all of them identified in pool THR-E (Fig. 2 and 3). Among them, only for Ttabunya1 could we find a second genomic segment (7.2-kb RNA1 and 1.2-kb RNA2) (Fig. 5B). Ttabunya2 has the longest genomic segment, with 10.2 kb (Fig. 5B), while Ttabunya3 and Ttabunya4 have very similar genome sizes, with 4.9 kb and 4.8 kb, respectively, that possibly represent incomplete virus segments.

Phylogenetic analysis (Fig. 9) of the identified *Bunyavirales* shows Ttabunya2, Ttabunya3, and Ttabunya4 clustering closely together with a bootstrap value of 100. Foaperi1 lies in a sister group of *Peribunyaviridae*, together with another soybean thrips-associated virus (16), indicating that the virus is only distantly related to the family *Peribunyaviridae* and that these viruses likely belong to a specific clade of thrips-infecting viruses. Ttabunya1 clusters with several insect bunyaviruses with a well-sustained bootstrap value, and Ttabunya4 can be found in a sister branch. Finally, Foanairo1 clusters in the orthonairovirus branch of the tree, supporting its relatedness to the *Orthonairovirus* genus, but it is basal to a group of insect-infecting nairo-like viruses that requires a possible new taxon.

One orthomyxo-like virus was identified in our data set and named insect metagenomic orthomyxo-like virus 1 (Imortho1) (Table 2 and Fig. 2). The genome is quadripartite (Fig. 5C), with 2,411 bases for RNA1, 2,263 bases for RNA2, 1,733 bases for RNA3, and 1,075 bases for RNA4. The RdRP domain is located on RNA2 and the nucleoprotein domain on RNA3, while RNA1 and RNA4 did not show any known domain. The four segments have been assigned to the same virus based on 5′ and 3′ end alignment (data not shown), which shows a high conservation in nucleotide sequences. The presence of the virus is limited to one pool (THR-E); moreover, the four genomic segments are present only in sample T9 (Fig. 3).

Phylogenetic analysis shows Imortho1 to be in a separate sister clade from taxonomically accepted orthomyxoviruses, indicating the need to define a new family to accommodate our virus and several others named orthomyxo-like viruses that have insects as true hosts (Fig. 9).

The bisegmented Frankliniella occidentalis associated qinvirus 1 (Foaqin1; putatively belongs to order *Muvirales*, family *Qinviridae*, genus *Yingvirus*) has been identified in a single sample (T7) of a unique pool (THR-D) with quite a high concentration based on read counts (Fig. 2) and qRT-PCR (Fig. 3). The results of qRT-PCR clearly show the presence of the two segments not only in the same pool but also in the same sample (T7) at a high concentration. Foaqin1 genomic organization (Fig. 5D) shows that RNA1 has one ORF coding for a putative RdRP, while RNA2 segment codes for two distinct ORFs, but they do not have conserved domains. Thrips tabaci associated yuevirus 1 (Ttayue1) shows a concentration similar, in terms of read counts, to that of Foaqin1 (Fig. 2). The genomic organization presents a bipartite genome with RNA1 coding for an RdRP and an RNA2 without any conserved domain (Fig. 5D). The conserved putative catalytic domain of Ttayue1 is SDD, corresponding to the yuevirus catalytic domain, while that of the qinvirus is IDD, the one present in Foaqin1 (data not shown).

Both the yueviruses and qinviruses characterized from invertebrates have a second genomic segment coding for a protein of unknown function, conserved among each of the two groups of viruses (14); indeed, we could find the second associated segment through a similarity search for the thrips qinvirus, while for the yue-like thrips virus, we could identify the second segment only through a careful analysis of ORFan sequences and correlation of abundance of reads mapping to distinct libraries and codetection of both segments in the same samples (Fig. 3).

Although the RdRP domain was not found in the ORF1 of Ttayue1, the BLAST hits are all RdRP (BLASTp program, database NCBInr limited to viruses [taxid 10239] in August 2020), so this sequence was used to generate the phylogenetic tree with RNA1 of Foaqin1, containing the RdRP domain (Fig. 9). The analysis placed the two viruses in two sister clades, each branch associated with a confirmed viral family: in the case of Foaquin1, we can support the taxonomical assignment to *Qinviridae* family, while Ttayue1 is only distantly related to previously described insect yuevirus and even more distantly related to the recently discovered yue-like mycoviruses (17).

### (v) Phylum *Duplornaviricota*.

**FIG 10:**
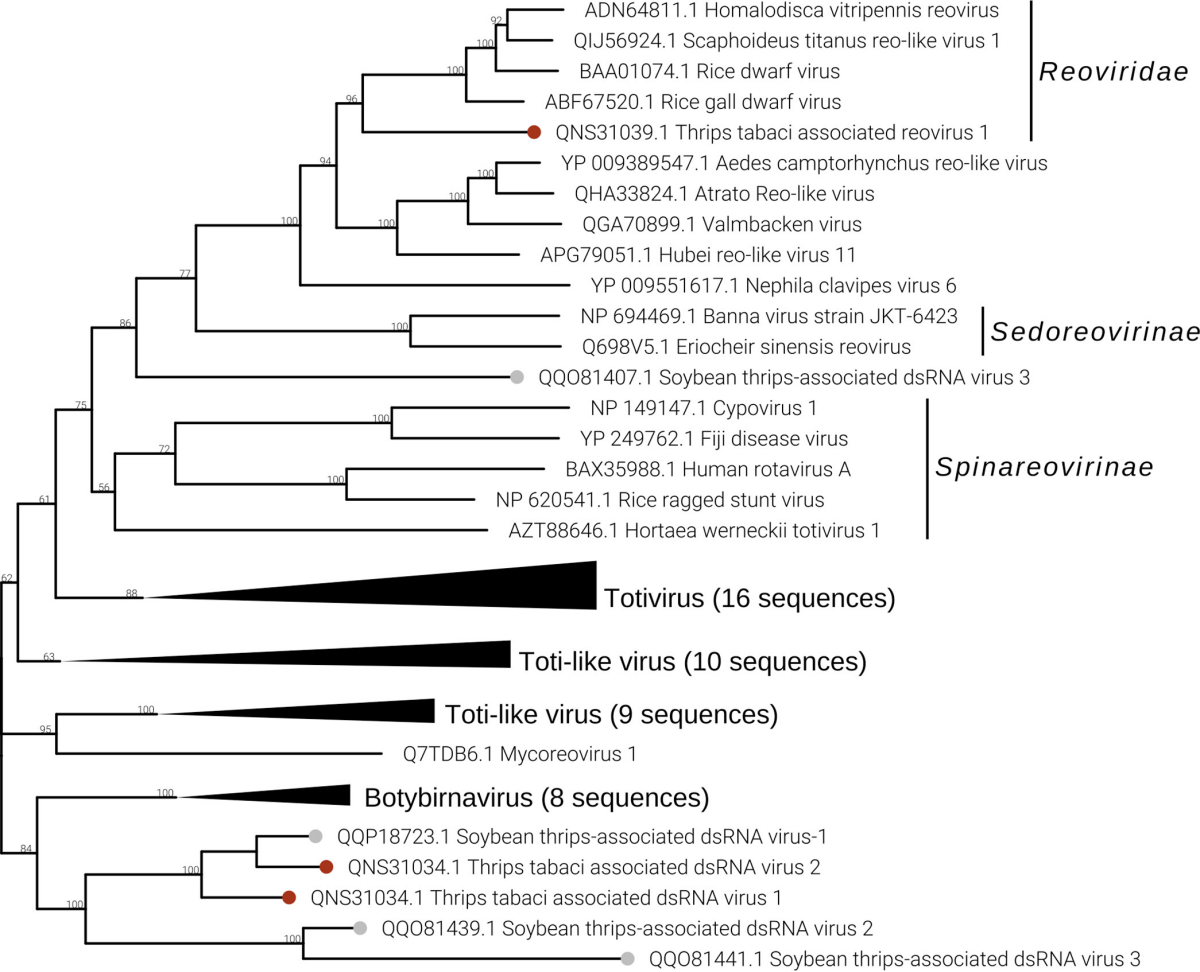
*Duplornaviricota* phylogenetic tree computed by IQ-TREE stochastic algorithm to infer phylogenetic trees by maximum likelihood. The model of substitution is VT+F+G4. The consensus tree is constructed from 1,000 bootstrap trees. The log likelihood of the consensus tree is −180450.432. At nodes are the percent bootstrap values. Different colors indicate different subgroups. Triangles indicate the insect metagenomics viruses, and circles indicate *Thrips tabaci*- or *Frankliniella occidentalis*-associated viruses. Viruses labeled with a gray circle are the ones identified in a recent soybean thrip virome characterization study (16).

In both sample pools THR-B and THR-E, we identified two double-stranded RNA (dsRNA) viruses named Thrips tabaci associated dsRNA viruses 1 and 2 (Ttads1 and Ttads2). The virus Ttads2 was in each pool, 10 times more expressed than Ttads1, based on read counts (Fig. 2), while the qRT-PCR amplified both viral contigs only in sample T9 (THR-E) (Fig. 3). Both viruses present an ORF2 coding for a putative RdRP protein with the closest BLAST hit to soybean thrips infecting dsRNA viruses (16), while the ORF1 does not contain any known domains based on MOTIF Search, but a BLAST search finds as first hit a proline-alanine-rich protein of Scaphoideus titanus toti-like virus 1 (NCBI:protein accession no. QIJ56902; 26% identity and 58% query coverage). The genomic organization is very similar to that of members of the genus *Totivirus*, presenting a putative −1 frameshift between ORF1 and ORF2 (Fig. 5E). The phylogenetic analysis showed the two viruses to be closely related to each other (Fig. 10). The closest virus on the tree is Scaphoideus titanus toti-like virus 1 (19), a toti-like virus recently identified in the Flavescence dorée phytoplasma vector Scaphoideus titanus (Ball). All the viruses present in the same clade, with a bootstrap value of 94, are viruses having an insect as a host.

Five segments of a single putative multisegmented reovirus have been identified in pool THR-E (data not shown). The virus, named Thrips tabaci associated reovirus 1 (Ttareo1), has the identified genome total size of 17,907 bp, with segment sizes ranging between 4,450 bp and 2,522 bp. Three conserved domains have been identified in the five segments, the RdRP on RNA1, the peptidase on RNA3, and the outer capsid protein P3 on RNA4 (data not shown). The last corresponds to the PF09231 PFam family, named Rice dwarf virus p3. Based on read counts, the five segments are only present in THR-E, apart from Ttareo1 RNA2, which seems to be also present in THR-B (Fig. 2); Ttareo1 RNA2 was amplified in sample T9 (Fig. 3).

Phylogenetic analysis (Fig. 10) of the Ttareo1 RdRP shows the virus clusters in a sister clade of the *Spinareovirinae* subfamily and in the same clade as the *Sedoreovirinae* subfamily, but on a deep branch, indicating that Ttareo1 can represent a member of a new *Reoviridae* subfamily.

### (vi) DNA virus.

In four pools out of five, we were able to detect the presence of a densovirus (Frankliniella occidentalis associated densovirus 1 [Foadenso1]) associated with *F. occidentalis*: pools THR-A, THR-C, and THR-D contain only the WFT, while THR-B contains a mix of WFT and OT (Fig. 2). Pool THR-B shows the lowest concentration of Foadenso1 reads count due to the sample contamination with OT. The qPCR results reveal a high concentration of Foadenso1 in T5 (*C_T_* of 24) but its absence in T8, the other sample in THR-B pool (Fig. 3).

Foadenso1 has a genome size around 5.5 kb (excluding the terminal inverted repeats, which we could not clone). The monopartite genome has four ORFs: ORF1 codes for a putative nonstructural protein (NS1), ORF2 codes for a putative viral late glycoprotein (BLLF1), also termed gp350/220, ORF3 codes for a putative densovirus capsid protein (Denso_VP4), and ORF4 has no conserved functional domain (Fig. 5F).

**FIG 11:**
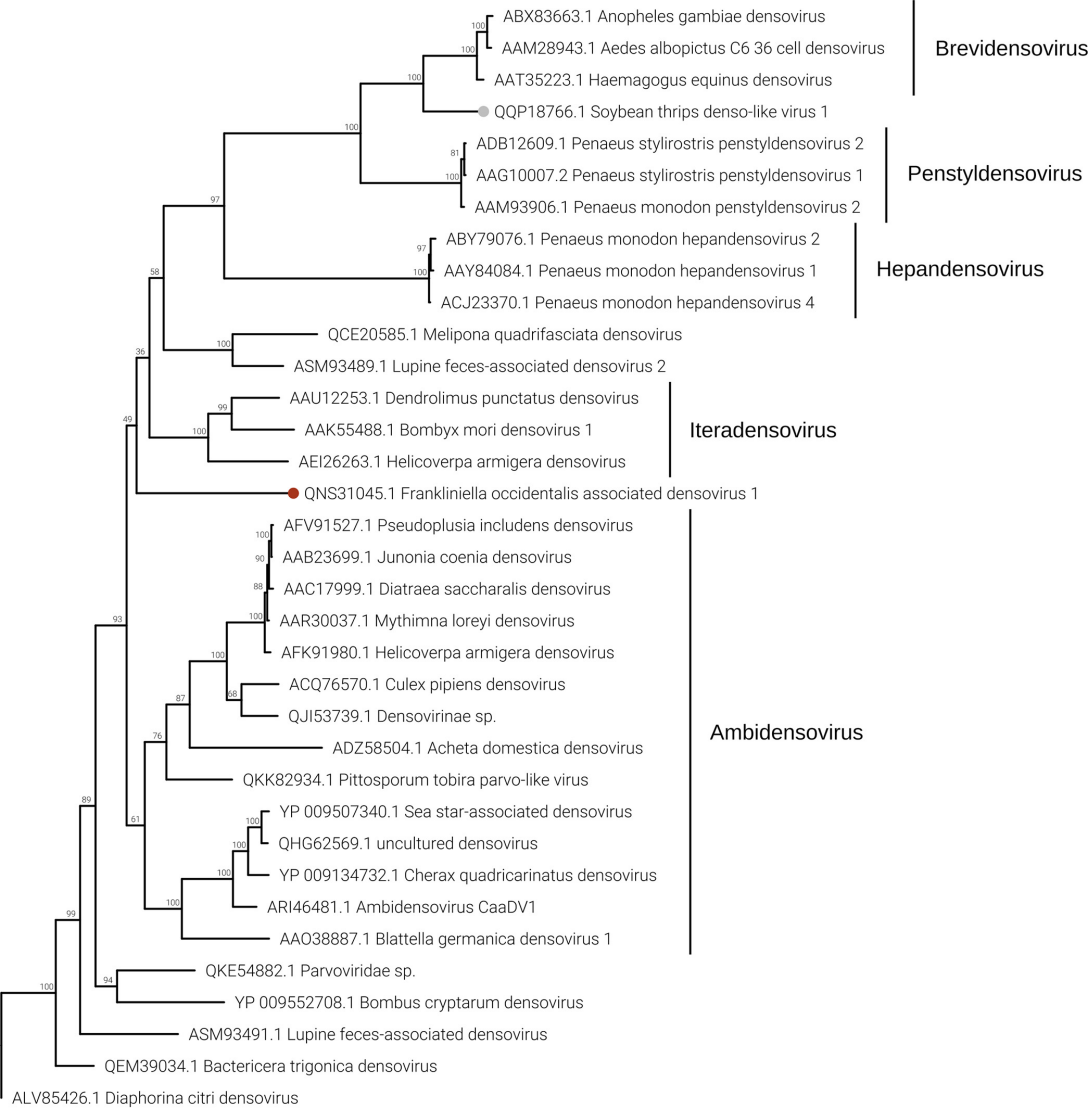
Densovirus phylogenetic tree computed by IQ-TREE stochastic algorithm to infer phylogenetic trees by maximum likelihood. The model of substitution is LG+F+I+G4. The consensus tree is constructed from 1,000 bootstrap trees. The log likelihood of the consensus tree is −35692.033. At nodes are the percent bootstrap values. A red circle shape is the label for the virus identified in this work. The virus labeled by a gray circle is the one identified in a recent soybean thrip virome characterization study (16).

Phylogenetic analysis of NS1 proteins (Fig. 11) shows Foadenso1 virus to be an outgroup to the *Densovirinae* subfamily clade. Also, the two closest homologues to Foadenso1, based on NCBI BLAST results (BLASTp, 2 August 2020)—Diaphorina citri densovirus and lupine feces-associated densovirus 2—are far from Foadenso1 and lie on a different branch of the tree with the real *Densovirinae* subfamily viruses. The phylogenetic tree seems to indicate that Foadenso1 is related only to the *Densovirinae* subfamily and can be a member of a new *Parvoviridae* subfamily. The other subfamilies of the *Parvoviridae* family—*Hamaparvovirinae* and *Parvovirinae—*are in different clades of the tree.

### Endogenized virus fragments.

Among the contigs we identified as viral, four contigs were amplified by PCR also from DNA, suggesting their possible integration in the genome of insect hosts (Table 1). BLAST analysis of the available full genomes of WFT, using as queries all the virus contigs we identified, confirmed the presence of the fragment corresponding to contig THR-D_DN18510, a fragment of a positive-strand virga-like virus, while fragment THR-B_DN27856 has partial hits in the *Thrips palmi* genome and is a fragment of a putative rhabdo-related sequence. Interesting, contrary to what has been reported so far in most analyses, where only small segments of the virus have been endogenized, here we report the assembly of a full-length iflavirus-like sequence, with high similarity to soybean thrips iflavirus 2 (contig THR-E_DN24098); its presence in only two populations of OT (data not shown) could hint at the possibility that the DNA we amplified corresponds to a partial cDNA resulting from host reverse transcriptase activity corresponding to a full-length replicating virus as observed in other entomovirus-host or mycovirus-host systems (24, 25).

### Persistence of viruses in specific thrips populations.

**FIG 12:**
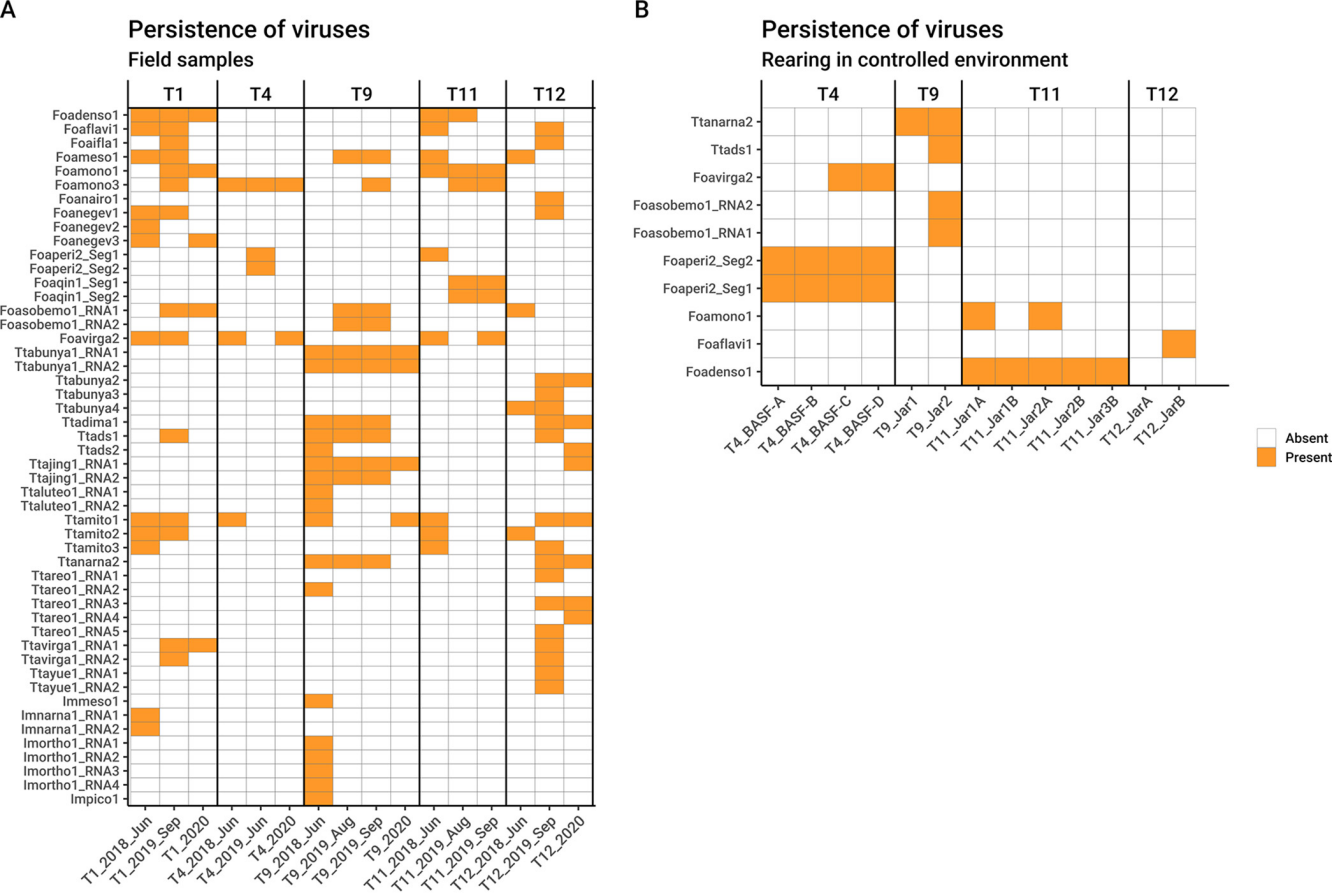
Graphical representation of qRT-PCR results showing the presence of each viral contig in each RNA sample. Above the heat map are indicated the sample names. (A) Virus persistence over the studied years; on the *x* axis are the years of sample collection. (B) Virus persistence in mass reared populations in controlled environment; on the *x* axis are the names of the rearing subpopulations.

**(i) Field populations.** To check the persistence of the identified viruses from WFT and OT populations in different years, some of the locations (fields) inspected in 2018 were sampled also in 2019 and 2020. Virus fragments were amplified using a qRT-PCR approach (qRT-PCR was used solely for detection and not for quantification). Figure 12A contains the correspondence between the 2018 samples and those found in the following years.

Few viruses were consistently detected over the 3 years (Foadenso1 in the field corresponding to sample T1, Foamono3 in the field corresponding to sample T4, Ttabunya1 in sample T9, and Foamono1 in the field corresponding to sample T11). As a representative example, in WFT from pepper in Piedmont (T1), 10 viruses were present in at least 2 years out of 3 and 7 were present in only 1 year. This mix of occasionally found and consistently found viruses in the same field over the years remains true also for the other four populations surveyed.

For the thrips population collected on onion in Jordan in 2019, results of qRT-PCR on all viruses of our 2018 library showed the presence of the Ttadima1, which in Italy was found in samples T9 and T12.

### (ii) Laboratory-reared populations.

Four populations sampled in 2019 (T12, T9, T11, and T4) were maintained in the laboratory and reared on healthy bean pods changed at each generation, without infestations from other insects, to assess the effect of living in captivity on the insect virome and to exclude that viruses detected in thrips might come from ingested plant sap, where a mix of other insects might feed under natural conditions. Results are summarized in Fig. 12B. In sample T4, Foaperi2 was found in the field in 2019 and was consistently detected in all four cohorts (i.e., individuals from the same rearing jar), confirming its persistent presence in WFT at high prevalence. In contrast, Foavirga2 was not detected in the 2019 field population but was then identified in rearing populations, but segregating among cohorts. Foadenso1 was present both in 2019 field samples and in all the cohorts tested from population T11; Foamono1 was amplified only in two cohorts out of five. The two T9 cohorts confirmed the presence of Ttanarna2 consistently, while Ttads1 and Foasobemo1 were present in only one of the two cohorts, confirming a tendency to segregate among individual thrips. Finally, sample T12 has lost all the viruses identified in the population from the field in 2019, except for Foaflavi1.

**FIG 13:**
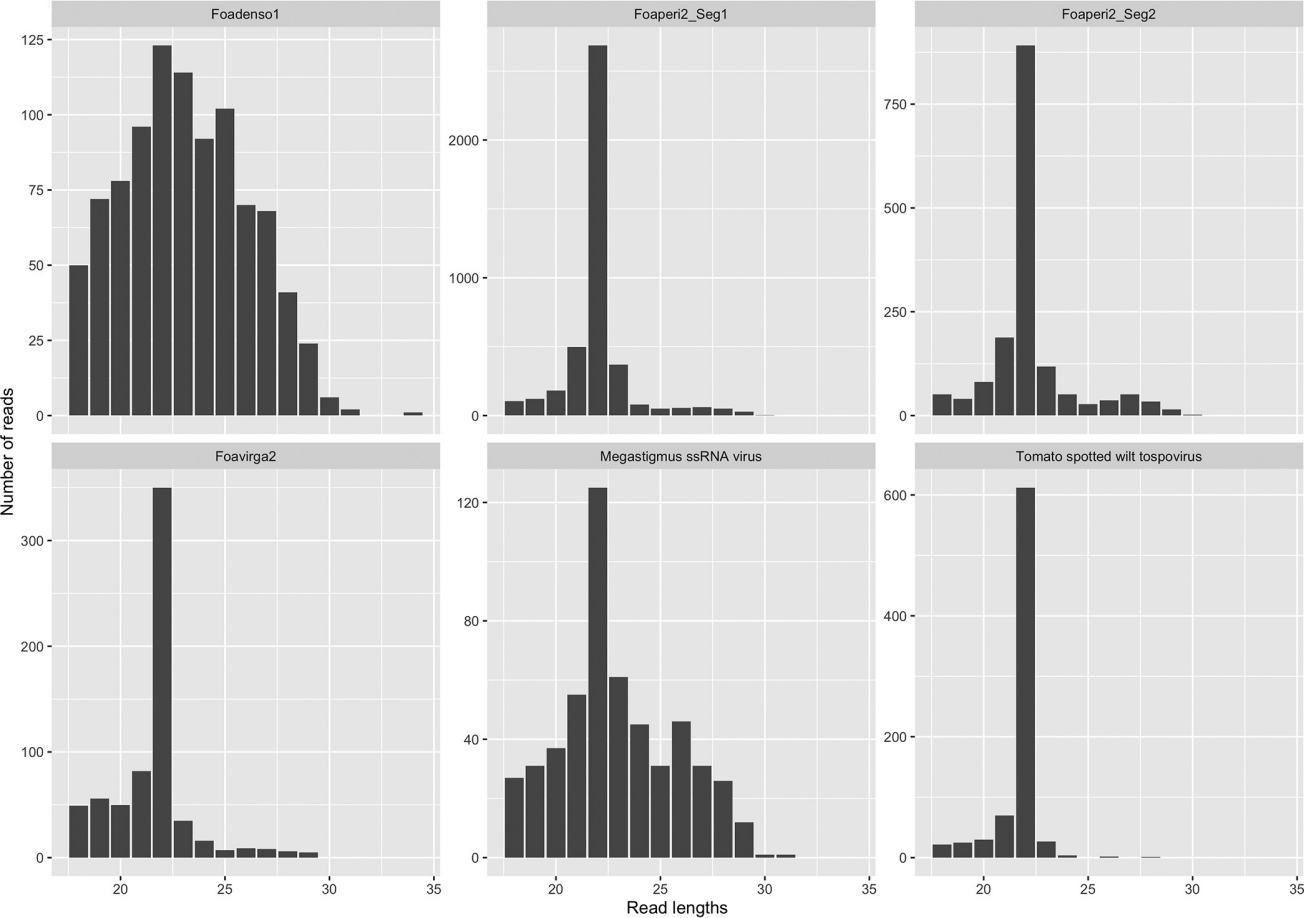
Small RNA read length distributions on selected viruses.

As a confirmation of the persistence of specific viromes in some populations, we report here the small RNA (sRNA) sequencing of a population reared in a controlled environment for two successive years, corresponding to sample T1 but collected in 2013 (Table 3 and Fig. 13): in that population, maintained over many generations, we could report the presence of both segments of the peribunya-like virus, the densovirus, and the Foavirga2 virus. Interestingly, the 22-nucleotide (nt)-long sRNA is the prominent size in length distributions of the sRNA mapping to replicating entomovirus with an RNA genome, while in the case of densovirus and the endogenized virus, lengths of sRNA are more uniformly distributed. A 26-nt secondary peak is also present. To our knowledge this is the first description of small RNA distribution of entomovirus from thrips (Fig. 13).

**TABLE 3:** Small RNA mapping results

Virus	Length of contig	No. of reads	NCBI hit	Host
Foaperi2_Seg1	9,618	4,290	Frankliniella occidentalis associated peribunyavirus-like virus 2 segm1	Thrips virus
Foaperi2_Seg2	4,897	1,587	Frankliniella occidentalis associated peribunyavirus-like virus 2 segm2	Thrips virus
Foadenso1	5,570	939	Frankliniella occidentalis associated densovirus 1	Thrips virus
Foavirga2	12,138	673	Frankliniella occidentalis associated virga-like virus 2	Thrips virus
THR-D_DN18510	1,167	529	Megastigmus ssRNA virus	Viral insertion
THR-D_DN18945	7,762	515	Tomato spotted wilt tospovirus S + M segment	Plant virus
THR-C_DN21959	8,918	278	Tomato spotted wilt orthotospovirus L segment	Plant virus
Foanairo1	4,933	45	Frankliniella occidentalis associated nairo-like virus 1	Thrips virus
Foaflavi1	19,679	31	Frankliniella occidentalis associated flavi-like virus 1	Thrips virus
T-Ame_DN4615	3,117	28	Ustilago maydis virus H1	Fungal virus
THR-E_DN20119	1,556	17	Iris yellow spot virus	Plant virus
T-Ame_DN13641	1,013	13	Ustilago maydis virus H1	Fungal virus
Foamono1	14,297	11	Frankliniella occidentalis associated mononegavirales virus 1	Thrips virus

## DISCUSSION

Insects were among the first organisms that were recognized as holobionts, particularly since some of them have specific organs that accommodate bacterial obligatory symbionts (26–28). The mutualistic symbiotic relationship between insects and a subset of their microbiota can be harnessed, resulting in detrimental effects on insect fitness or in their vectoring capacity. A striking example of a translational application of such approach is the recently reported successful artificial infection of the hemipteran planthopper *N. lugens* with a *Wolbachia* strain (wStri) from Laodelphax striatellus (Fallén): the new bacterial association with the insect host inhibited both infection and transmission of rice rugged stunt virus, therefore offering a clear proof of concept for a new strategy to prevent plant virus infection through alteration of the insect vector microbiota (11). Such a possible approach in agriculture follows previous successful approaches to interfere with mosquito-borne arbovirus (particularly dengue flavivirus) through *Wolbachia*-based population replacement or population suppression strategies (29–31).

In this respect, the microbiota associated with insects is not limited to bacteria, and insect-specific viruses (ISV) are also persistently present in insect populations (32, 33). The possibility of exploiting this specific interaction to prevent arbovirus transmission (again, mostly of human-infecting flaviviruses) has been envisioned and has been shown to be based on superinfection exclusion (34); an example of successful interference with transmission is that of the ISV Nhumirim virus blocking West Nile virus transmission in mosquitoes (35). These premises encouraged us to pursue the first step (virome characterization) of a similar strategy for the containment of thrips and the viruses they vector because of the need for new approaches to limit their great direct and indirect economical damage (36).

The different genomic components of the thrips holobiont are beginning to be unveiled: recently, the genome of WFT and that of the melon thrips (*T. palmi*) were characterized (37, 38); the bacteria associated with thrips were also partially characterized in the case of WFT (39–41). Regarding the viral component of the thrips microbiome, knowledge is still very limited: a recent work described viruses associated with soybean thrips samples from various U.S. regions (16); furthermore, four genomovirus genomes were amplified from Echinothrips americanus (Morgan), a pest thrips sampled in Florida (42). Here, we investigated the virome associated with two important thrips vectors, WFT and OT, with samples collected mostly in Italy, where WFT is an invasive species of Nearctic origin introduced in the late 1980s (43), whereas OT is endemic to the Mediterranean basin (7).

Considering important vectors of plant pathogens (bacteria, phytoplasmas, and viruses), the viromes associated with some important species have been reported (19, 44–47).

Our work includes samples mostly from Italy (with only two samples from the United States and one from Jordan), and this allowed us to show variation in resident viromes even in a relatively small geographic area (samples inside Italy were collected less than 1,000 km apart). We found more viral species in *T. tabaci* (even if with a smaller number of samples), and this could be due to bottlenecks associated with the recent invasion of the WFT that could explain its lower diversity than of an endemic species (OT); nevertheless, we cannot see the dramatic reduction of viruses associated with other invasive species sampled in newly invaded areas (19, 48).

Our work describes new viruses that are diverse enough from existing viruses to warrant new higher-rank taxonomic recognition: a first example is the different genomic organization of the mesoni-like viruses characterized from thrips in comparison with those previously characterized from other insects (18).

Another interesting new virus deserving further taxonomic attention is most closely related to the recently characterized orfanplasmoviruses (17) tentatively named insect metagenomics narna-like virus 2: the virus we found was very abundant in two WFT populations from Piedmont and Sicily. We could find for the first time a second RNA associated with the orfanplasmo-like virus through a further search in the ORFan protein-coding segments from our libraries. However, we searched also Plasmopara viticola assemblies from our previous work (17) by BLAST using as a query the putative protein encoded by RNA2, but we were not able to retrieve homologues, suggesting that they are too distant to be retrieved by homology searches (a contention supported by the little similarity also present at the RdRP level). The presence of narna-like viruses in metazoans (other than in fungi and apicomplexan), including insects, was recently confirmed (49–51).

Even if we cannot exclude that the mito-like and narna-like viruses we included as likely insect-associated viruses could be indeed fungal viruses, their relatively high abundance in some samples and the very low abundance of true mycoviruses and fungal reads suggest that they could indeed be entomoviruses.

The tombus/sobemo-like viruses we identified are also new entomoviruses belonging to clades that need a taxonomic classification and that have not been characterized biologically or ecologically.

The same lack of biological characterization is also true for the numerous virga-like viruses from insects that constitute new virus clades; one of them (Foavirga2) is present in our laboratory-reared populations, allowing us to attempt a more complete biological characterization in the near future. The negev-like viruses we found are instead a completely new virus clade representing a distinct phylogenetic branch, possibly thrips specific, that requires taxonomic accommodation. A negev-like virus was also found in the virome of soybean thrips (16), but the genome is much shorter (9.3 kb, compared to over 14 kb) than those of the WFT-infecting negev-like viruses.

The jingmen-like virus for which we characterized two segments belongs to a well-established clade of insect-infecting multipartite viruses (opposed to the original tick-infecting jingmen viruses): the best characterized of these viruses is the multisegmented Guaico culex virus (52).

Insect-infecting negative-strand viruses are very common and have been well characterized from many insect taxonomic groups (15). Among those putatively infecting thrips, a knowledge gap that needs to be filled is related to the characterization of the putative peribunya-like virus from *F. occidentalis*, a virus that is persistent in a population we reared in a controlled environment for many years and that can be found in populations in the field. All the closely related viruses were characterized from metagenomic studies: in fact, only two segments were identified, and a putative nucleocapsid protein was never identified, not even for Ixodes scapularis bunyavirus, a virus that was characterized from a tick cell line (53). Lack of a nucleocapsid is unlikely, since in *Bunyavirales* the nucleocapsid plays a role in replication.

Other expanding groups of negative-strand RNA viruses are those in the order *Muvirales* (qin-like viruses) and in the order *Goujianvirales* (yue-like viruses). To our knowledge, none of the qin-like or yue-like insect viruses has been characterized biologically, and given the relatively high accumulation in some thrips populations, we could suggest thrips as a good model system to study them.

The only DNA virus we found in our thrips metatranscriptome is a densovirus-like sequence that was always associated with some WFT populations in Italy. This virus is basal to a still-not-well-characterized group of insect-infecting denso-like viruses, among which the best characterized is the Diaphorina citri densovirus (54). We could not find evidence of the presence of an ssDNA genomovirus-like sequence as shown instead for *E. americanus* (42).

In general, our work has shown that viruses infecting thrips are mostly species specific. In fact, even if the populations of WFT and OT we sampled are in some cases geographically very close (a few kilometers apart), there is only limited, unconfirmed evidence of common viruses between the two species (as an example, the Foamesoni1 in WFT populations T1, T4, and T11 and OT populations T9 and T12). Also, we can for the first time show a regional specificity of viromes in a thrips species. Some of these viruses are likely part of a “core virome,” since we can see their high titer after rearing in controlled environment over time, as in the case of Foperi2, Foadenso1, and Foavirga2. All three viruses were present in a population from pepper from Piedmont which we have used in a number of leaf disk assays in 2012-2013 (55, 56) and that are still present in the same area, after almost 10 years.

We developed an interactive map available at https://thrips-virusmap.herokuapp.com/ as a webpage or at https://hub.docker.com/repository/docker/chiapellom/virusmap as a docker image that allows one to see the geographical distribution of each virus in our set of samples over time. We also have preliminary evidence that some viruses are maintained over time in populations reared in controlled environments, whereas some other are lost: in analogy to what has been demonstrated for *Aedes* species (57), we also can hypothesize the more constant presence of cryptic viruses that can occasionally be integrated by pathogenic viruses.

The value of insect virome characterization through next-generation sequencing (NGS) for surveillance of viruses of vertebrates and plants potentially threatening their health has been underlined previously (16): in our case, we also found evidence of plant viruses of interest, but none of them is new for the locations monitored, in contrast to new orthotospovirus and new tenuivirus found in soybean thrips samples (16). The presence of putative mycoviruses in our samples can be due to fungi being part of the thrips-associated microbiome (no specific study has investigated fungi in thrips), or to the presence of entomopathogenic fungal species in some individuals, but since the discovery that also some partiti-like sequences can directly infect insects (58), we cannot disregard the possibility that some of them are indeed true thrips viruses.

Given the recent publications of the virome associated with another thrips species (ST) from populations in 8 states in the United States in 20 different locations (16), we can compare the results: given the different size of the area surveyed and the number of samples for a total of more than 15,000 individual thrips in the ST study and around 4,000 in our 2018 survey, the total numbers of virus contigs identified in the two studies are similar (181 contigs for ST and 95 in our study). Nevertheless, the only common viral sequence is indeed related to the single sample of ST in our study, where the same sobemo-like sequence was identified; we associated with this virus a second RNA, which escaped detection in the work from Thekke-Vetil and collaborators (16). Therefore, even in this case, we have no evidence of cross-species infection among three distinct species: in the comparison with ST, the distant geographical area surveyed could also play a role. Nevertheless, some commonalities among the thrips viromes so far characterized can be drawn: in fact, we also have more plus-strand RNA viruses than minus-strand and dsRNA viruses, and, finally, the same class of DNA virus is the least represented one (denso-like viruses). Furthermore, phylogenetic analysis shows that some clades are indeed enriched of thrips viruses, showing some commonalities among the different viruses infecting thrips possibly resulting in thrips-specific clades.

A number of studies have shown different competence in tospovirus transmission comparing different populations of the same vector species (59–62), pointing to great variability that likely has a strong genetic component as previously shown both in the thrips vector (63–65) and the tospovirus (55, 66). An additional hypothesis is that interactions with a resident virome could also play an important role in different vectoring capacity: in fact, the coinfection of two viruses in the same insect could be synergistic, antagonistic, or neutral. Both antagonistic and synergistic relationships can result in lower tospovirus transmission, either because of direct interference with tospovirus accumulation (antagonism) or because synergism can tip the balance of neutrality of single infection toward detrimental effects and population collapse when one or both viruses increase their titer considerably. A greater titer in itself might not explain specific local interactions as recently reported for TSWV-OT interaction (67). Other finely tuned molecular interactions playing different roles in distinct insect immune pathways could also come into play, as recently shown for Argentine ants (68), and this should be studied on a case-by-case virus-virus interaction.

## MATERIALS AND METHODS

### Preliminary field collection of thrips populations in 2018.

An initial sampling effort was organized in Italy and, to a lesser extent, in the United States during summer 2018. Thrips populations were sampled in seven Italian regions (Piedmont, Veneto, Liguria, Emilia Romagna, Campania, Puglia, and Sicily) spanning from north to south and from west to east. Thrips populations (200 to 250 individuals) were collected from a variety of crops: tomato (2 samples), leek (2 samples), onion (2 samples), pepper (2 samples), strawberry (2 samples), white African daisy/*Dimorphoteca* sp. (1 sample), and watermelon (1 sample). Sampling was performed by scouting flowers and leaves on a white tray (250 by 350 mm) and transferring the fallen adults into glass vials (diameter, 24 mm; length, 120 mm) with an insect aspirator. In some cases, the whole plants were collected in plastic bags and further processed in the laboratory as described above. In total, 12 individual field populations were collected (Table 4). A subsample of 30 adult thrips belonging to each population was examined under a stereomicroscope for their identification (OT or WFT). Two populations were sampled from the United States (pool T-ame): one from a field (ST) and one from a greenhouse (WFT).

**TABLE 4:** Sample collection

Sample	Thrips species	Plant host	Region	Pool
T4	*F. occidentalis*	Watermelon	Emilia Romagna	THR-A
T6	*F. occidentalis*	Tomato	Campania	THR-A
T10	*F. occidentalis*	Tomato	Puglia	THR-A
T5	*F. occidentalis/Thrips tabaci*	Onion	Veneto	THR-B
T8	*Thrips tabaci*	Onion	Piedmont	THR-B
T1	*F. occidentalis*	Pepper	Piedmont	THR-C
T2	*F. occidentalis*	Strawberry	Veneto	THR-C
T3	*F. occidentalis*	Pepper	Veneto	THR-D
T7	*F. occidentalis*	Pepper	Sicily	THR-D
T11	*F. occidentalis*	Dimorphoteca	Liguria	THR-D
T9	*Thrips tabaci*	Leek	Liguria	THR-E
T12	*Thrips tabaci*	Leek	Piedmont	THR-E
T13	*Neohydatothrips variabilis*	Green bean	USA (lab)	T-ame
T14	*F. occidentalis/Thrips tabaci*	Soybean	USA (field)	T-ame

### Field collection and laboratory rearing of selected thrips populations in 2019-2020.

In summer 2019 and 2020, thrips populations (500 to 1,000 individuals) were collected in five locations corresponding to five samples from 2018—T1 (WFT, pepper, Piedmont), T4 (WFT, watermelon, Emilia-Romagna), T9 (OT, leek, Liguria), T11 (WFT, *Dimorphoteca*, Liguria), and T12 (OT, leek, Piedmont)—and a new one was added from Jordan (OT, onion, Jordan Valley). Sampling was performed as described above. All field-collected adults were cold anesthetized and identified under a stereomicroscope; only adults that belonged to the species *F. occidentalis* or *T. tabaci* were used for further analyses and mass rearing. To maintain populations in purity in a controlled environment, mass rearing was established on a subset of these populations, starting from at least 300 adults for every field-collected population. Laboratory populations were reared on green pea pods, Pisum sativum (L.), inside 1-liter glass jars in growth chambers at 20 to 26°C with a 16-h photoperiod. Every 2 to 3 days some green pea pods were replaced, and granulated pollen was regularly added as a dietary supplement.

### Total RNA extraction and sample pooling.

Total RNA from each thrips population was extracted using TRIzol as previously described (55). RNA extracts were quantified with a NanoDrop 2000 spectrophotometer (Thermo Scientific, Waltham, MA). To reduce sequencing costs, the RNA extracted from individual populations was pooled to have five final samples with the same final concentration (7 ng/μl).

### RNA sequencing and bioinformatics pipeline.

After rRNA depletion with Ribo-Zero Gold human/mouse/rat (Epicentre Biotechnologies, Madison, WI), cDNA library preparation (Illumina Inc., San Diego, CA; TrueSeq Stranded) and sequencing were carried out by Macrogen Inc. (Seoul, Republic of Korea).

Bioinformatics workflow was divided into five main steps: (i) read quality check and filtering, (ii) assembly of clean reads into contigs, (iii) identification of viral sequences, (iv) “uni-contig” production, and (v) mapping of reads on the viral genomes. Step i was performed using BBTools (v 38.70) (69), in order to remove Illumina adaptor sequences and artifacts and short and residual ribosomal sequences. In step ii, clean reads were used as input for Trinity software (v 2.3.2) (70) for *de novo* assembly. In step iii, DIAMOND (v 0.9.21.122) (71) was used to perform a match between assembled contigs and a custom viral database using BLASTx (taxonomy identifiers for viruses used to create the custom viral database are txid1925802, txid1921431, txid1917979, txid1915204, txid1922240, txid2732900, txid2732416, and txid2732396). After visual inspection of alignments obtained with BLASTx, selected candidate viral contigs were aligned by BLAST against the NCBI nonredundant protein database (version September 2020) to discriminate between already discovered viruses, host sequences present in viral genomes, and true new virus sequences. CAP3 (default parameters) (72) was used to further assemble selected contigs. Step iv aimed to reduce the redundancy of viruses present in different libraries. After concatenating the contigs from all libraries, individual contigs from each library were aligned by BLAST against it. Results were processed, and contigs with identity over 90% and length over 1,000 nucleotides were grouped and considered a single virus contig in our final list. The longest contig of each group (named “uni-contig”) was selected as the representative contig and deposited in NCBI. Uni-contig selection was performed by custom R scripts. Step v was performed using bowtie2 (73) and a custom R script.

### ORFan contig detection.

Each assembled library was aligned with DIAMOND against the NCBI nonredundant whole database (version September 2020). Every contig with a significative BLAST hit was discarded, while the contigs without an NCBInr BLAST hit, longer than 1 kbp, and encoding a protein of at least 15 kDa were kept. This set of contigs defined the “ORFan” sequences. ORFan contigs were mapped with reads considering their orientation, and contigs that showed only positive or only negative reads were discarded, since a typical feature of replicating viruses is the presence of both minus- and plus-sense genomic template for replication (17).

### Genome organization.

The NCBI ORF finder tool (https://www.ncbi.nlm.nih.gov/orffinder/) was used for open reading frame prediction, while MOTIF Search (https://www.genome.jp/tools/motif) was used for functional domain search. Default parameters were applied for both software.

### Taxonomic analysis of the metasample.

In order to evaluate the taxonomic complexity of the metasamples present in our libraries, we used Kraken2 (74) in combination with Pavian (75) and a custom R script to create visualization plots.

### Phylogenetic analysis of viral sequences.

RNA-dependent RNA polymerase (RdRP) proteins from all identified viruses and at least the closest 10 homologues from NCBI databases were used for phylogenetic analysis. Representative viruses, based on International Committee on Taxonomy of Viruses (ICTV) classification, were also added when needed to support the subgroups in the phylogenetic tree. The phylogenetic analysis was performed using ggtree R package (76). Msa R package (10.18129/B9.bioc.msa) was used to align the sequences using the MAFFT algorithm with default parameters (77). The RdRP alignments were then processed by IQ-TREE software (78) to obtain phylogenetic trees using the default parameters (combine ModelFinder, tree search, ultrafast bootstrap, and SH-aLRT test) and the maximum likelihood (ML) model. Treeio and ggtree R packages were used for tree data import, manipulation, and display (76, 79). The accession numbers of the proteins and the corresponding virus names and acronyms are displayed on the trees.

### Virus names.

Viruses identified in this paper have been named using the following criteria: (i) the first part of the name is the main taxon present in the metasample source of the virus, or when such assignment is not reconfirmed by further more specific assays, the term “insect associated” was used; (ii) the second part of the name identifies the virus taxonomic group, if the virus taxonomic assignment was clear; and (iii) the last part of the name is a sequential number. For example, the contig THR-A_DN23655 has as first match Artashat orthonairovirus and the corresponding virus was named Frankliniella occidentalis associated (part i) nairo-like virus (part ii) 1 (part iii).

### Association between thrips viruses and specific thrips samples by qRT-PCR.

Real-time reverse transcription-quantitative PCR (qRT-PCR) analyses with virus-specific primers (see Table S1 in the supplemental material) were performed to associate any specific virus assembled *in silico* with the original specific RNA sample (a cohort of five thrips/sample). DNA copies were produced using a high-capacity cDNA reverse transcription kit (Thermo Fisher Scientific, Waltham, MA) following manufacturer instructions. qRT-PCR was performed using a CFX Connect real-time PCR detection system (Bio-Rad Laboratories, Hercules, CA) and iTaq Universal SYBR Green Supermix (Bio-Rad Laboratories). The same primer pairs specific for each virus contig were used in qPCR to check for possible endogenization events using total nucleic acids as the template.

### Small RNA high-throughput sequencing from a WFT population.

A population of WFT, initially sampled from pepper in Piedmont in 2014, from the same field corresponding to sample T1, was maintained in purity until 2017. Total RNA was extracted as described above, and small RNAs were purified and sequenced by the BGI Group company as described previously (80). Reads were mapped to the viral contigs resulting from our virome characterization as described above. Size distribution of the sRNA reads was displayed as described previously (80).

### Data availability.

All raw reads have been deposited in the Sequence Read Archive (SRA) under BioProject number PRJNA637687 and BioSample numbers SAMN15150576 to SAMN15150580. All the insect-associated viral contigs have been deposited in GenBank under accession numbers MN714662 to MN714667, MN714669 to MN714672, MN714674 to MN714683, MN714686 to MN714690, MN725049 to MN725052, MN764138 to MN764161, MN787040 to MN787042, MW297844 to MW297846, MW459158, and MW459159 (Table 2). Script and fasta files of clean small RNA reads are available at the following link: https://bit.ly/3m9Yw5Q.
