# Supplementary material for: Complexity and Local Specificity of the Virome Associated with Tospovirus-Transmitting Thrips Species
Source: J Virol. 2021 Oct 13;95(21):e00597-21. doi: 10.1128/JVI.00597-21 (PMC8513489; doi:10.1128/JVI.00597-21)
Supplement: Supplemental file 1 — Table S1. Download JVI.00597-21-s0001.pdf, PDF file, 0.2 MB [file jvi.00597-21-s0001.pdf]

**Supplementary Table 1**. Oligonucleotide sequences of the primers used for specific amplification of virus segments through qRT-PCR.

| virus | Primer | Sequence | Organism |
|---|---|---|---|
| Foadenso1 | F | CCCTGTGGAGTCACTTCGTC | Frankliniella occidentalis associated densovirus 1 |
| Foadenso1 | R | CACGACGGTACTGCCCTATC | Frankliniella occidentalis associated densovirus 1 |
| Foaflavi1 | F | GGCTGCGTAGGATCTTCTCC | Frankliniella occidentalis associated flavi-like virus 1 |
| Foaflavi1 | R | GTCGATCAGTTGCGCATGAC | Frankliniella occidentalis associated flavi-like virus 1 |
| Foaifla1 | F | GGTTACCAACAAACACGCCC | Frankliniella occidentalis associated iflavirus 1 |
| Foaifla1 | R | CCGAGGTTTCGGATGTGGAA | Frankliniella occidentalis associated iflavirus 1 |
| Foameso1 | F | CGTCGACGGCGTAATCAGTA | Frankliniella occidentalis associated mesonivirus 1 |
| Foameso1 | R | GCAGGTGGCTGAACCAGTAT | Frankliniella occidentalis associated mesonivirus 1 |
| Foamono1 | F | CTGTATGACTCCCATCGGGC | Frankliniella occidentalis associated mononegavirales virus 1 |
| Foamono1 | R | GCGAACATCTCAATGGCGAC | Frankliniella occidentalis associated mononegavirales virus 1 |
| Foamono2 | F | AACGGAGGGTTACCGCAAAT | Frankliniella occidentalis associated mononegavirales virus 2 |
| Foamono2 | R | TCCTGGTTCGAGGATTCCCT | Frankliniella occidentalis associated mononegavirales virus 2 |
| Foamono3 | F | GGCAGCTGACACTCCATTCT | Frankliniella occidentalis associated mononegavirales virus 3 |
| Foamono3 | R | CCGCCCTTCTAAGCAAGTCA | Frankliniella occidentalis associated mononegavirales virus 3 |
| Foanairo1 | F | CGAGGACTCTGGACAGTTGG | Frankliniella occidentalis associated nairo-like virus 1 |
| Foanairo1 | R | CCAAGGTCAGTGAAGTGCCA | Frankliniella occidentalis associated nairo-like virus 1 |
| Foanarna1_RNA1 | F | ATCACGTGAGTTGGGACGAC | Frankliniella occidentalis associated narna-like virus 1 RNA1 |
| Foanarna1_RNA1 | R | GATGCACCAAACCCGTAGGA | Frankliniella occidentalis associated narna-like virus 1 RNA1 |
| Foanegev1 | F | TATTCAGTTGGGGACGGCAC | Frankliniella occidentalis associated  negev-like virus 1 |
| Foanegev1 | R | CGTCGCGAGTCTTATCGGAA | Frankliniella occidentalis associated  negev-like virus 1 |
| Foanegev2 | F | CTGCAGCATGCTTTGGTTGT | Frankliniella occidentalis associated  negev-like virus 2 |
| Foanegev2 | R | TACTACCAGCCGCACAGTTG | Frankliniella occidentalis associated  negev-like virus 2 |
| Foanegev3 | F | CATGGGCAACTTGCGAACAA | Frankliniella occidentalis associated negev-like virus 3 |
| Foanegev3 | R | CAATATCCCGCGCATCCTCT | Frankliniella occidentalis associated negev-like virus 3 |
| Foaperi2_Seg1 | F | TGGAAAGAACCGTGCAAAGC | Frankliniella occidentalis associated peribunyavirus-like virus 2 segm1 |
| Foaperi2_Seg2 | R | ACGGTGTTGAGCTTCCTAGC | Frankliniella occidentalis associated peribunyavirus-like virus 2 segm1 |
| Foaperi2_Seg2 | F | TGCCACTCATTTGCAGACCT | Frankliniella occidentalis associated peribunyavirus-like virus 2 segm2 |
| Foaperi2_Seg2 | R | CAACATCAGGCAGACTGGGT | Frankliniella occidentalis associated peribunyavirus-like virus 2 segm2 |
| Foaqin1_RNA1 | F | ACGCAGGGAATTGTCCATGT | Frankliniella occidentalis associated qin-like virus1 RNA1 |
| Foaqin1_RNA1 | R | GAACCACACCCTGTCTCCAG | Frankliniella occidentalis associated qin-like virus1 RNA1 |
| Foaqin1_RNA2 | F | CCAGGCAGAGCGTCCATTAT | Frankliniella occidentalis associated qin-like virus1 RNA2 |
| Foaqin1_RNA2 | R | ACTCAGGCCCAGACGATACT | Frankliniella occidentalis associated qin-like virus1 RNA2 |
| Foasobemo1_RNA1 | F | GACACGCTTCAGAGGTTGGA | Frankliniella occidentalis associated sobemo-like virus 1 RNA1 |
| Foasobemo1_RNA1 | R | TCTGTGAACTCATGCCCGAC | Frankliniella occidentalis associated sobemo-like virus 1 RNA1 |
| Foasobemo1_RNA2 | F | TGGCACCCATTCCTCAATCC | Frankliniella occidentalis associated sobemo-like virus 1 RNA2 |
| Foasobemo1_RNA2 | R | TCCGAAAGAGTGCCTTGCAT | Frankliniella occidentalis associated sobemo-like virus 1 RNA2 |
| Imsobemo2_RNA1 | F | CCAGGAGTCAACTCGCCTTT | Insect metagenomics sobemo-like virus 2 RNA1 |
| Imsobemo2_RNA1 | R | ATGCTCCTGTGTTTAGGGGC | Insect metagenomics sobemo-like virus 2 RNA1 |
| Imsobemo2_RNA2 | F | TCAATGTCACCCTTCGGTGG | Insect metagenomics sobemo-like virus 2 RNA2 |
| Imsobemo2_RNA2 | R | CCAGAGTCCCAAGAACCGTC | Insect metagenomics sobemo-like virus 2 RNA2 |
| Foavirga1_RNA1 | F | TGGTACAACGGGTTCACGAG | Frankliniella occidentalis associated virga-like virus 1 RNA1 |
| Foavirga1_RNA2 | R | AGCAGGCAGGTTTAGTCGTC | Frankliniella occidentalis associated virga-like virus 1 RNA2 |
| Foavirga1_RNA1 | F | TCGCCCATGGTCAGACAAAA | Frankliniella occidentalis associated virga-like virus 1 RNA1 |
| Foavirga1_RNA2 | R | ACTCCCTCACCGATAACCGA | Frankliniella occidentalis associated virga-like virus 1 RNA2 |
| Foavirga2 | F | AAGAGCCAAATCCGCGTACA | Frankliniella occidentalis associated virga-like virus 2 |
| Foavirga2 | R | CAGAGTCAGGTGCAGAAGGG | Frankliniella occidentalis associated virga-like virus 2 |
| Ttabunya4 | F | GCACTATGTCACCAACTGCT | Thrips tabaci associated bunya-like virus 1 |
| Ttabunya4 | R | TGGCTTCAGAATCGTTGTCTCT | Thrips tabaci associated bunya-like virus 1 |
| Ttabunya1_RNA1 | F | GGAAGTGAGACACGGCTTGA | Thrips tabaci associated bunyavirales 1 RNA1 |
| Ttabunya1_RNA1 | R | TGAGAGGGATCCTCCCCTTG | Thrips tabaci associated bunyavirales 1 RNA1 |
| Ttabunya1_RNA2 | F | AGCTGGGCTTTGTAGGTTCC | Thrips tabaci associated bunyavirales 1 RNA2 |
| Ttabunya1_RNA2 | R | ACGGCGATCCAGGAGATAGT | Thrips tabaci associated bunyavirales 1 RNA2 |
| Ttabunya2 | F | CCTGGACCAGTTAGTGCTGC | Thrips tabaci associated bunya-like virus 2 |
| Ttabunya2 | R | TCCCTCATTCCACCCCATTG | Thrips tabaci associated bunya-like virus 2 |
| Ttabunya3 | F | CACGCAGGATCCCAACTTCT | Thrips tabaci associated bunya-like virus 3 |
| Ttabunya3 | R | GCTTGTGCATCATCACTGTGT | Thrips tabaci associated bunya-like virus 3 |
| Ttadima1 | F | AGCTCTTCACTCTTCCGCAC | Thrips tabaci associated dimarhabdovirus 1 |
| Ttadima1 | R | GCCTACAGGTCAGCAACCTT | Thrips tabaci associated dimarhabdovirus 1 |
| Ttads1 | F | TAAGGGCATGATGGCTGCAA | Thrips tabaci associated dsRNA virus 1 |
| Ttads1 | R | CCAACAGGCTTAGGGAGCAA | Thrips tabaci associated dsRNA virus 1 |
| Ttads2 | F | CCTTCCATCTGGGCAACAGT | Thrips tabaci associated dsRNA virus 2 |
| Ttads2 | R | GCAAGCTTCATTCGCCGTAG | Thrips tabaci associated dsRNA virus 2 |
| Ttajing1_RNA1 | F | ATTTCCGCTGTTGGTCGAGT | Thrips tabaci associated jingmen-like virus 1 RNA1 |
| Ttajing1_RNA1 | R | TCCGTGAGGTTGAGTTCTGC | Thrips tabaci associated jingmen-like virus 1 RNA1 |
| Ttajing1_RNA2 | F | CGATGCTATGCATTCTGCCG | Thrips tabaci associated jingmen-like virus 1 RNA2 |
| Ttajing1_RNA2 | R | CGTAGTCCTTGGCTGCAAGA | Thrips tabaci associated jingmen-like virus 1 RNA2 |
| Ttaluteo1_RNA1 | F | TGGTCCTTGGTTTTGGGCTT | Thrips tabaci associated luteo-like virus 1 RNA1 |
| Ttaluteo1_RNA1 | R | GATGTGCAGCAAACAACCGT | Thrips tabaci associated luteo-like virus 1 RNA1 |

| | | | |
|---|---|---|---|
| Ttaluteo1_RNA2 | F | CCGCTCAAAGGGCTTACAGA | Thrips tabaci associated luteo-like virus 1 RNA2 |
| Ttaluteo1_RNA2 | R | ATGACCCTCTCCCGACTTGA | Thrips tabaci associated luteo-like virus 1 RNA2 |
| Ttameso1 | F | AGTAGGTAGTCCGAAGGCGT | Thrips tabaci associated mesonivirus 1 |
| Ttameso1 | R | CAAGGCATTGTCGTACTGCG | Thrips tabaci associated mesonivirus 1 |
| Ttamito1 | F | ATTGGATGCAGTGTGAACGC | Thrips tabaci associated mitovirus 1 |
| Ttamito1 | R | AGTGAGTTGTTCAGAGTGAGC | Thrips tabaci associated mitovirus 1 |
| Ttamito2 | F | AAAGGGTGCTCGTTTGGAGG | Thrips tabaci associated mitovirus 2 |
| Ttamito2 | R | GTGACTTCATAGCCTGCCGT | Thrips tabaci associated mitovirus 2 |
| Ttamito3 | F | GATTTGAAAGGTTGTCCCGCT | Thrips tabaci associated mitovirus 3 |
| Ttamito3 | R | TGTTACACAACTTCGGGATGC | Thrips tabaci associated mitovirus 3 |
| Ttanarna1 | F | AAGACCGATCAAGCATGGCA | Thrips tabaci associated narna-like virus 1 |
| Ttanarna1 | R | GTGGCCTCCTTCAAATCCGA | Thrips tabaci associated narna-like virus 1 |
| Ttanarna2 | F | AATCCTTGCTCCGCCATCAA | Thrips tabaci associated narna like virus 2 |
| Ttanarna2 | R | TTCGCGTTACCTTCTCCGAC | Thrips tabaci associated narna like virus 2 |
| Ttaortho1_RNA1 | F | TTCACGCGGATGTGTTCTCA | Thrips tabaci associated orthomyxo-like virus 1 RNA1 |
| Ttaortho1_RNA1 | R | TGCCAGTCAACTTGGAGCAT | Thrips tabaci associated orthomyxo-like virus 1 RNA1 |
| Ttaortho1_RNA2 | F | TGGAATCGGACCTGGGAGAT | Thrips tabaci associated orthomyxo-like virus 1 RNA2 |
| Ttaortho1_RNA2 | R | GCCTTGAGGTTTCTGAGCCT | Thrips tabaci associated orthomyxo-like virus 1 RNA2 |
| Ttaortho1_RNA3 | F | CGCCAAAATAGCTCCACACG | Thrips tabaci associated orthomyxo-like virus 1 RNA3 |
| Ttaortho1_RNA3 | R | TCCTCGGAGGTGCAACAATC | Thrips tabaci associated orthomyxo-like virus 1 RNA3 |
| Ttapico1 | F | TGTTATCTTTGCAGCGGGGT | Thrips tabaci associated picorna-like virus 1 |
| Ttapico1 | R | CAGGTCGAACCAGACGTCAA | Thrips tabaci associated picorna-like virus 1 |
| Ttatombus2_RNA1 | F | GGTGTGAGGGTCTTGGATGG | Thrips tabaci associated tombusbipa-like virus 1 RNA1 |
| Ttatombus2_RNA1 | R | ACCGTAACGTGCTCAGAAGG | Thrips tabaci associated tombusbipa-like virus 1 RNA1 |
| Ttatombus2_RNA2 | F | CCATACTCGTGCCTACCACC | Thrips tabaci associated tombusbipa-like virus 1 RNA2 |
| Ttatombus2_RNA2 | R | GTGCAAACTGCTGTGAAGGG | Thrips tabaci associated tombusbipa-like virus 1 RNA2 |
| Ttavirga1_RNA1 | F | TGCTATGCAAGAGGTGGTGG | Thrips tabaci associated virga like virus 1 RNA1 |
| Ttavirga1_RNA1 | R | AGTTGTGCCGTAACCCTCTG | Thrips tabaci associated virga like virus 1 RNA1 |
| Ttavirga1_RNA2 | F | CACTCCGATGTGACAGGCTT | Thrips tabaci associated virga like virus 1 RNA2 |
| Ttavirga1_RNA2 | R | AAATGCACAGCTGCTTTGGG | Thrips tabaci associated virga like virus 1 RNA2 |
| Ttavirga2 | F | CGTCGTCCCAGACAGATACG | Thrips tabaci associated virga like virus 2 |
| Ttavirga2 | R | AAAGGTCAGTTGCTCGGAGG | Thrips tabaci associated virga like virus 2 |
| Ttayue1_RNA1 | F | CGCGGGAATAGCTGAGTCTT | Thrips tabaci associated yue-like virus 1 RNA1 |
| Ttayue1_RNA1 | R | AGGCTGGTACGGATCCTCTT | Thrips tabaci associated yue-like virus 1 RNA1 |
| Ttareo1_RNA1 | F | TTGGTATGCCTCCCTCGGTA | Thrips tabaci associated reovirus 1 RNA1 |
| Ttareo1_RNA1 | R | CTCAGTTGGCGTGAGCCTTA | Thrips tabaci associated reovirus 1 RNA1 |
| Ttareo1_RNA2 | F | CCAACTGCCTTCTCAAACGC | Thrips tabaci associated reovirus 1 RNA2 |
| Ttareo1_RNA2 | R | ATAGTCCACCCAAATGGCGG | Thrips tabaci associated reovirus 1 RNA2 |
| Ttareo1_RNA3 | F | CGGTTTCAAAACACCGCACT | Thrips tabaci associated reovirus 1 RNA3 |
| Ttareo1_RNA3 | R | CAGCGGTAATGAATCGGGGA | Thrips tabaci associated reovirus 1 RNA3 |
| Ttareo1_RNA4 | F | CACCCCTCGAAGTCCTTGAC | Thrips tabaci associated reovirus 1 RNA4 |
| Ttareo1_RNA4 | R | TGAGACCTCACGTTTCACCG | Thrips tabaci associated reovirus 1 RNA4 |
| Ttareo1_RNA5 | F | TCGTTCAATGACCCTCCACG | Thrips tabaci associated reovirus 1 RNA5 |
| Ttareo1_RNA5 | R | AACGCTCAACACATCGTTGC | Thrips tabaci associated reovirus 1 RNA5 |
| Ttayue1_RNA2 | F | TCCTTCGACAGGCCATTCAC | Thrips tabaci associated yue-like virus 1 RNA2 |
| Ttayue1_RNA2 | R | TCCTGCAGGTGCATTGCTAA | Thrips tabaci associated yue-like virus 1 RNA2 |
| Ttaortho1_RNA4 | F | GCATGCAGTGAAACCTAGCG | Thrips tabaci associated orthomyxo-like virus 1 RNA4 |
| Ttaortho1_RNA4 | R | AAGCCCAACGTCGCTAATGA | Thrips tabaci associated orthomyxo-like virus 1 RNA4 |
| Foanarna1_RNA2 | F | CCCCGACGATCCACACTATG | Frankliniella occidentalis associated narna-like virus 1 RNA2 |
| Foanarna1_RNA2 | R | AACAGTCTGGCGTTCTAGCC | Frankliniella occidentalis associated narna-like virus 1 RNA2 |
| Ttatombus1 | F | TTCACTGAAAGGGGACGACG | Thrips tabaci associated tombus-like virus 1 |
| Ttatombus1 | R | CGCGGCTAGTTAGTGGGTAG | Thrips tabaci associated tombus-like virus 1 |
